# The economic benefits of reducing cardiovascular disease mortality in Quebec, Canada

**DOI:** 10.1371/journal.pone.0190538

**Published:** 2018-01-04

**Authors:** David Boisclair, Yann Décarie, François Laliberté-Auger, Pierre-Carl Michaud, Carole Vincent

**Affiliations:** 1 HEC Montréal, Montréal, Québec, Canada; 2 Institut national de la recherche scientifique (INRS) - Urbanisation, culture et société, Montréal, Québec, Canada; 3 Consultant in Social and Economic Studies, Montréal, Québec, Canada; Scientific Institute of Public Health (WIV-ISP), BELGIUM

## Abstract

**Objectives:**

We assess how different scenarios of cardiovascular disease (CVD) prevention, aimed at meeting targets set by the World Health Organization (WHO) for 2025), may impact healthcare spending in Quebec, Canada over the 2050 horizon.

**Methods:**

We provide long-term forecasts of healthcare use and costs at the Quebec population level using a novel dynamic microsimulation model. Using both survey and administrative data, we simulate the evolution of the Quebec population’s health status until death, through a series of dynamic transitions that accounts for social and demographic characteristics associated with CVD risk factors.

**Results:**

A 25% reduction in CVD mortality between 2012 and 2025 achieved through decreased incidence could contain the pace of healthcare cost growth towards 2050 by nearly 7 percentage points for consultations with a physician, and by almost 9 percentage points for hospitalizations. Over the 2012–2050 period, the present value of cost savings is projected to amount to C$13.1 billion in 2012 dollars. The years of life saved due to improved life expectancy could be worth another C$38.2 billion. Addressing CVD mortality directly instead would bring about higher healthcare costs, but would generate more value in terms of years of life saved, at C$69.6 billion.

**Conclusions:**

Potential savings associated with plausible reductions in CVD, aimed at reaching a World Health Organization target over a 12-year period, are sizeable and may help address challenges associated with an aging population.

## Introduction

As the leading cause of death in adult Canadian men and women, cardiovascular disease (CVD) ranked first in 2008 for direct health costs. Those reached nearly C$12 billion in 2008[1]. In 2013–2014, heart attacks and heart failure ranked third and fourth, respectively, among reasons for hospitalization. When measured in average length of hospital stay, heart failures ranked first, with 9.2 days, while heart attacks were third with 5.1 days [2]. A review of the evidence in the U.S., Australia and Canada concluded to a significant economic burden from CVD [3]. The economic burden of CVD has also been found to be large in the U.S. (at $457 billion, in 2004 US dollars) and in Europe (at $169 billion) [4] [5].

There has been a steady and marked decline in CVD prevalence over the past decades, especially among the older population, and the largest gains in life expectancy at age 60 have come in part from the reduction in CVD mortality. In high-income countries such as the U.S. or Canada, lower CVD and diabetes prevalence has led to a gain of about 3 years in life expectancy for men and 4.3 years for women between 1980 and 2011[6]. More recently, the policy focus has shifted towards ways to reduce the incidence of these diseases through primary and secondary prevention. While some studies have shown limited efficacy of multi-dimensional risk interventions [7], others argue that new approaches show promise, in particular those focusing on major risk factors among the young [8]. Irrespective of the approaches or interventions chosen, the World Health Organization adopted in 2013 a target of reducing worldwide chronic disease (including CVD) mortality by 25% by 2025 [9].

Most economic burden assessments of CVD use static approaches (i.e. they focus on one year). Instead, we use a dynamic microsimulation model, recognizing various pathways to CVD, disability and mortality, to project major categories of healthcare spending in Quebec to the year 2050. We assess how different scenarios of reduced CVD incidence or mortality, based on targets set by the World Health Organization for 2025, may affect the costs of hospitalizations and consultations with physicians. We simulate in great detail the evolution of the health status of each individual until death, through a series of dynamic transitions that accounts for social and demographic characteristics associated with CVD risks as well as the different links between the incidence, prevalence and mortality associated with several major disease groups. We provide long-term forecasts of healthcare use and costs at the population level based upon microlevel data from surveys and administrative sources.

## Methods

We project the health of individuals aged 30 and over for the population of Canada from 2012 to 2050 using COMPAS, a health-oriented microsimulation model—whose code and full technical documentation are available in a public repository [10]–recently developed following a framework developed for the United States [11]. Although projections can be made for the entire Canadian population, we focus on the province of Quebec for which we were able to obtain administrative data on the cost of hospital stays and physician compensation. Healthcare funding is for the most part the responsibility of provincial governments in Canada, with some portion of the financing coming from the Federal government. We then build alternative scenarios regarding the future evolution of CVD, and compute for each scenario 1) healthcare cost savings for physician visits and short-term hospital stays; 2) increases in life expectancy and years of life saved; and 3) the monetary value of the flow of healthcare cost savings and life expectancy increases discounted over the projection period. For the latter exercise we use a real discount rate of 3% and a value of $200,000 per life-year saved [12].

### Study population, model structure and characteristics

Simulations are done in four stages in COMPAS. First, the base population is created using Statistics Canada’s 2010 Canadian Community Health Survey, which contains a cross-section of over 60,000 records of individuals living in private households, including 11,000 in Quebec. Records are weighted to ensure that the base population is representative of the Canadian population in 2010. Each person in the base population is characterized by various socio-demographic dimensions (gender, age, educational attainment, immigrant status); health status (diagnosis of diabetes, hypertension, stroke, cancer, heart disease, lung disease or dementia); major risk factors (tobacco use and obesity); and disabilities (activity limitations and cognitive impairment). We define someone has having CVD if he has one of the following self-reported doctor diagnoses: heart disease, stroke and hypertension.

In a second stage, we compute the probability that an individual will change his or her health status over the following two-year period. Developing a disease, being admitted into a long-term care facility, starting or quitting smoking, and dying are examples of changes in health status that are modelled jointly—i.e. transition probabilities differ for every combination of possible states for the health status variables. Econometric modelling is used to estimate the transition probabilities—or incidence—for each of the seven diseases considered in the model, as a function of age, major risk factors, other socio-demographic characteristics (gender, education attainment, immigrant status) and, in certain clinically plausible cases, the prior presence of other diseases in an individual. Complementary log-log models are applied to data from Statistics Canada’s National Population Health Survey (1994–2011) to estimate these transition probabilities. Assumptions about whether the presence of some illnesses is allowed to affect the incidence of others are based on medical research and were validated by a panel of experts as part of the Future Elderly Model (FEM) initiative [13]. Information about these assumptions—as well as the data and all econometric models used—are provided in chapter 4 of the technical appendix (S1 File), as are details about the modelling of mortality as well as back-casting and validation of the transition models.

In a third stage, administrative data from Quebec’s single-payer healthcare system and data from the National Population Health Survey are used to project care use and related costs for each individual. The survey’s longitudinal sample includes 17,276 Canadians from all ages. Respondents were first interviewed in 1994–1995, and the same individuals were interviewed every two years until 2010–2011. Through econometric modelling described in the technical appendix (S1 File), mostly two-part models explaining whether use is positive and then the amount of use conditional on use, we estimate relationships between an individual’s health status and personal characteristics and the type and quantity of medical resources used in the previous 12 months. Three medical resource categories are modelled for this article: the number of consultations with a generalist (e.g. family doctor, paediatrician or general practitioner); the number of consultations with a specialist; and the number of nights spent in a hospital (excluding long-term care facilities or convalescent homes). As found in other studies looking at the direct healthcare cost effect of CVD, these three sources may account for up to 80% of total direct cost; the remaining 20% is mainly due to drug spending, which is not available to us for this study [14].

We estimate the cost of hospitalizations using MED-ECHO, a database that documents all hospital stays in every Quebec hospital that provides general and specialized care. Information includes the exact length of stay, the principal and secondary diagnoses that were made, the hospital service in which the individual stayed and the types of medical interventions received. All costs are estimated in constant 2012 Canadian dollars. For every year up to 2050, a—declining—“structural cost” growth factor is applied: 1.5% annually over the period from 2013 to 2018, 1% per year from 2018 to 2028, 0.5% from 2028 to 2038 and 0% thereafter. This factor reflects the fact that over recent decades, growth in spending has exceeded what could be accounted for by known factors such as aging.

Administrative data on consultation billing by general practitioners and medical specialists are used to estimate the costs of each consultation. Data indicates whether the consultation took place in a hospital setting. That allows us to include the costs of consultations with a physician taking place at the hospital in the cost of hospitalizations rather than including them separately in the cost of consultations with a physician. For each individual, we combine the information about all consultations to determine the annual number of consultations with a physician (generalists and specialists separately).

In the final stage, new cohorts of individuals enter the simulation in each period at the starting ages of 30 and 31. Stages 2,3 and 4 are then repeated for every 2-year period, and the last cohort is added in year 2050. The model is almost entirely based on population dynamics: it tracks individuals as they move from one two-year period to the next. In each simulation cycle, the population loses some individuals due to mortality (or because they reached the maximum allowed age of 110) and gains others. The model also incorporates recent trends in tobacco use, obesity and educational attainment, so that the simulated future population reflects secular changes in these variables—in general, we prolong past 10-year trends one decade into the future, subsequently cut them by half every decade, and set them to zero for 2040–2050 (see chapter 5 of the technical appendix (S1 File) for details). We also incorporate assumptions about how the Quebec population will change over time, as described in the technical appendix (S1 File)–e.g. with respect to immigration, size of entering cohorts, and exogenous improvements in overall mortality, which are all set to match Statistics Canada’s most recent projections [15]. At any given time, the simulated population is representative of the Quebec population aged 30 and over.

Because of the randomness associated with these simulations, 50 replications of the projection process are performed for each of the scenarios with 100 sets of possible values for the parameters of the underlying econometric models (100 bootstrap samples are used to estimate transition model parameters). Hence, a total of 5,000 replications are performed, and the estimates obtained are averaged over those replications. We also report the 5^th^ and 95^th^ percentiles of those simulations.

### Three scenarios

We construct three projection scenarios, which differ according to the assumptions made about the evolution of CVD among future cohorts of Quebec residents. Our first scenario establishes a baseline: we assume no medical improvements and no change in the behavior and lifestyle habits that could have an impact on the ways individuals transition into a new health status. In this scenario, the risks of developing a disease are thus constant over time for a given set of personal characteristics, including health status.

Under the baseline scenario, which reflects the natural future evolution of all variables as computed by the model, COMPAS projects that in 2050 the Quebec population will reach 10.2 million. This projection stands between the medium-growth and the high-growth projection scenarios published by the Quebec statistical agency [16]. Life expectancy at ages 30 and 65 generated by COMPAS (54.8 and 21.7 years, respectively) is also very much in line with official projections [15], as shown in Table 4.6 of chapter 4 in the technical appendix (S1 File).

We build our alternative scenarios using the World Health Organization’s target #1 for 2025 [9]. We model two different pathways to reaching the target, which includes a 25% reduction in CVD mortality by 2025. The two pathways explored both account for the interaction between CVD incidence and mortality at the individual level. Because COMPAS does not model cause of death, we interpret the target as a 25% decrease in the age-adjusted death rate of individuals suffering from CVD (i.e., the number of individuals with CVD dying in a given period as a share of total population). Furthermore, because Canada already has a relatively good standing in terms of CVD prevention and care, we apply the target for the whole population 30 years and older, instead of the 30–70 y.o. population. We do not model specific interventions which may reach those targets, but rather assess the overall benefits that could be obtained from reaching such targets.

In a first alternative scenario, we decrease the death rate of individuals suffering from the two CVD groups that most often lead to death: heart disease and stroke. We decrease the death rates to reach a total decrease of 25% between 2012 and 2024 (COMPAS has 2-year simulation cycles). We proceed by calibration to find the appropriate decrease in death rates required to achieve the target, leaving the incidence of the diseases otherwise unchanged. Because the baseline scenario already involves a 12.7% age-adjusted decrease over that period, a future downward trend consistent with some of the literature for England [17], the additional decrease required in this “mortality-based scenario” is 16%–the sum exceeds 25% because of the cumulative effect of lowering mortality over time. This scenario can be construed as one of “treatment” on individuals already suffering from CVD, since no action is taken on the incidence of CVD. Such a decrease might be achieved, inter alia, through exercise rehabilitation for individuals suffering from coronary heart disease [18].

We also consider a second scenario where reduced CVD incidence is the main pathway to lower death rates in 2025 for individuals with CVD—thus leaving mortality risk unchanged for those individuals who suffer from CVD. This scenario might be thought of as oriented more toward primary prevention—for instance through improved use of statins, as suggested by some studies [19–20]. Even though in the case of coronary heart disease, some argue that the goal of preventing 90% of cases is now achievable [21], we only seek to reach the WHO target, via a reduced incidence of heart disease, stroke and hypertension. In order to reach a 25% reduction in deaths by 2024, we calibrate as in the first scenario and find that a 28% decrease in incidence, applied progressively between 2012 and 2022, is required to meet the target in this “incidence-based” or “prevention” scenario.

## Results

Projections from the baseline scenario yield an increase in the proportion of individuals suffering from CVD among those aged 30 and over. From 2012 to 2050, the prevalence of hypertension increases from less than 29% to almost 41%. The prevalence of heart disease also increases, from 7.6% in 2012 to more than 9.5% in 2050. The proportion of individuals projected to experience the effects of a stroke increases slightly but this result is not robust to model uncertainty as a significant number of runs result in a negative trend. The foregoing stands in contrast to the previous downward trends observed in Quebec and elsewhere, and is in part due to a composition effect associated with population aging and higher prevalence of chronic conditions among younger cohorts. We report our results about costs and life years saved in difference with respect to the baseline scenario.

### Healthcare costs

We estimate and report healthcare use and related costs under each scenario. Figures are computed in 2012 constant Canadian dollars. Cost levels in 2012 are normalized to 100.

**Fig 1:**
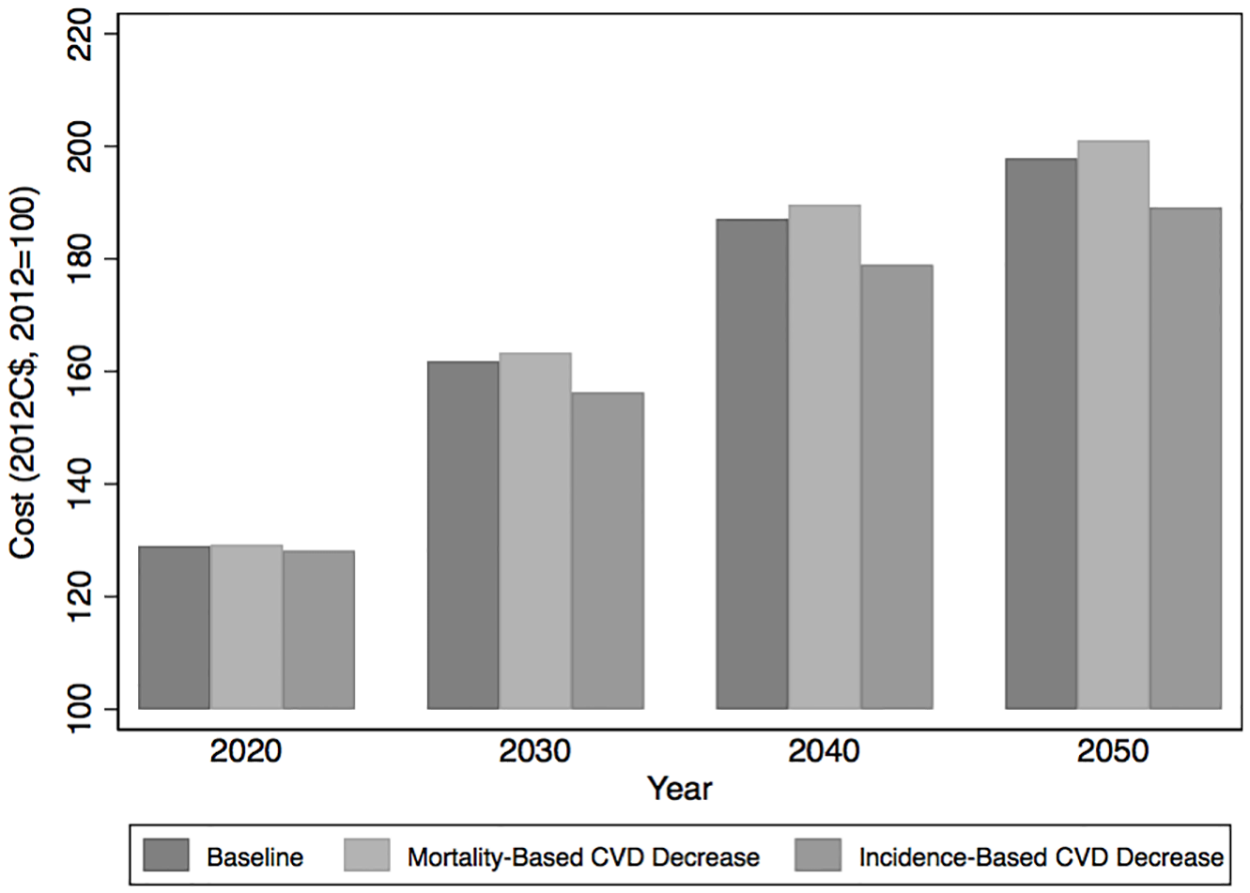
Cost of hospitalizations for Quebec, Canada, 2012 to 2050 (2012C$, 2012 = 100). Notes: Aggregate cost is normalized to 100 in the base year (2012) in the baseline scenario.

Hospitalizations account for the largest share of health spending related to CVD. Fig 1 shows projected trends in the costs of hospitalization, including the costs of consultations with a physician (generalist or specialist) within a hospital setting. A 25% reduction in CVD mortality achieved through decreased CVD incidence contains the growth in costs by nearly 9 percentage points over the period, from 97.8% under the baseline scenario to 89.1% (80.9; 97.1). In contrast, the growth in costs is *greater* in all periods under our mortality-based scenario, at 101% (92.7; 110.7), implying that this scenario will not generate hospitalization cost savings. This result is robust to model uncertainty, with 98% of parameter values used generating hospitalization cost increases.

**Fig 2:**
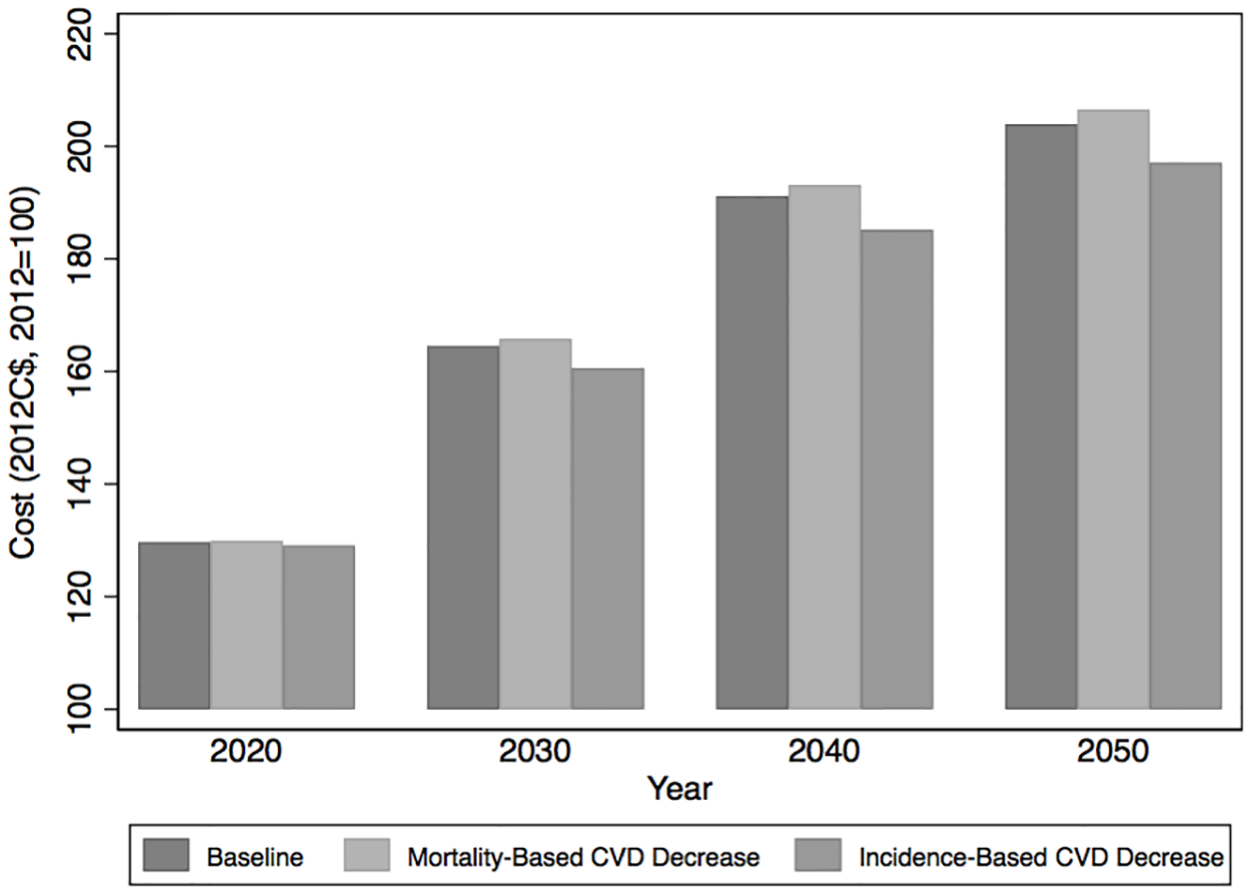
Cost of consultations with a physician (generalist or specialist) for Quebec, Canada, 2012 to 2050 (2012C$, 2012 = 100). Notes: Aggregate cost is normalized to 100 in the base year (2012) in the baseline scenario.

Fig 2 shows important increases in the flow of discounted future costs of consultations with physicians outside of a hospital setting—with a relationship between scenarios identical to the case of hospitalizations. Costs increase by nearly 104% by 2050 in the baseline scenario (97.1; 111.9), but the incidence-based scenario lowers the increase by almost 7 percentage points (-11.0; -4.0). The mortality-based scenario again here increases the costs of physician consultations by more than under the baseline in 2050 (106.5% [98.5; 116.1] instead of 103.9%). This scenario thus entails higher overall healthcare costs, which is expected because individuals with CVD remain alive for longer with their diseases. This, it should be noted, abstracts from the cost of any actual intervention that would bring about the changes modelled in the two scenarios, resulting only from the health and population dynamics in COMPAS.

The projected increases differ by physician category, with costs for visits to specialists increasing by 7 to 8 percentage points more in all scenarios (shown in the disaggregated data underlying Fig 2, available in our public repository [10]), but the difference *between* scenarios is similar—i.e. costs for both categories of physicians react similarly to the two alternative scenarios. The cost differences with respect to the baseline are robust to model uncertainty for physicians, except for the cost of visits to specialists, which exhibits a positive value 73% of the time.

### Life expectancy and years of life gained

Life expectancy is a crucial component of health improvements and is projected to increase over the projection period under all scenarios considered. Table 1 shows life expectancy at age 65, an age that is common to compute life expectancy and after which many—though not all—gains in life expectancy may be expected to occur from lower CVD mortality. Under the baseline scenario, life expectancy goes from 20.5 years in 2012 (19.7; 21.5) to 21.7 in 2030 (20.6; 22.8) and then 23.9 in 2050 (23.0; 25.0). The mortality-based scenario yields 0.3 additional year of life (0.001; 0.5) per person at age 65 for the whole Quebec population in 2050. The incidence-based scenario yields a similar result, but with smaller and slower increases in life expectancy—and in this case, 93% of our model runs yield a positive increase in life expectancy at 65.

**Table 1:** Life expectancy at age 65 for Quebec, Canada, 2012 to 2050 (years for the baseline; additional years compared to baseline for other scenarios).

Scenario	2012	2030	2050
*Baseline*	*20*.*5 (19*.*7; 21*.*5)*	*21*.*7 (20*.*6; 22*.*8)*	*23*.*9 (23*.*0; 25*.*0)*
-25% CVD Mortality by 2025 via Decreased Mortality	0.0 (-0.2; 0.2)	+0.3 (0.1; 0.5)	+0.3 (0.0; 0.5)
-25% CVD Mortality by 2025 via Decreased Incidence	0.0 (-0.2; 0.2)	+0.2 (0.0; 0.4)	+0.2 (0.0; 0.5)

Notes: Confidence intervals at the 5^th^ and 95^th^ percentiles are shown in brackets. “CVD Mortality” refers to all-cause mortality for individuals with a self-reported diagnosis of heart disease or stroke in the previous 2-year cycle.

Although the increase in life expectancy appears modest, the corresponding number of life-years saved at the population level is significant and large. As shown in Table 2 for ages 30 and older (COMPAS does not directly project the population under 30), by 2050, the scenario where CVD reduction occurs primarily through reduced mortality saves a total of 753,300 years of life (461,700; 1,008,700)–and nearly 40,000 for 2050 only. Even by 2030, only 5 years after the reduction target is reached, 119,300 years of life have been saved (32,000; 211,000). The “incidence-based” reduction scenario also brings about a large number of life years saved, at 433,200 (192,200; 680,200). But interestingly, by 2030 this scenario would possibly not save a significant number of years of life, as indicated by the confidence interval. This result highlights the fact that disease reductions that operate through a decreased incidence—as opposed to decreased mortality for all those suffering from the disease—yield mortality results that take longer to materialize.

**Table 2:** Years of life gained after 30 y.o. in Quebec, Canada, 2012 to 2050 (in thousands), under two CVD reduction scenarios.

Scenario		2030	2050
-25% CVD Mortality by 2025 via Decreased Mortality	Annual gain	19.7 (10.5; 28.6)	39.2 (24.9; 51.6)
	Cumulative gain	119.3 (32.7; 211.0)	753.3 (461.7; 1,008.7)
-25% CVD Mortality by 2025 via Decreased Incidence	Annual gain	8.0 (-0.1; 15.8)	27.4 (14.3; 41.1)
	Cumulative gain	44.7 (-42.2; 140.6)	433.2 (192.2; 680.2)

Notes: Confidence intervals at the 5^th^ and 95^th^ percentiles are shown in brackets. “CVD Mortality” refers to all-cause mortality for individuals with a self-reported diagnosis of heart disease or stroke in the previous 2-year cycle.

### Monetary value of healthcare cost savings and of years of life saved

Using aggregate expenditure data on all hospitalizations and physician consultations for the Quebec population aged 30 and over, we can project future flows of healthcare cost savings achieved over the 2012–2050 period in our two alternative scenarios, with respect to the baseline (or, in one case, the additional healthcare costs, or negative savings). To do so, we apply to these aggregate figures the growth rates predicted by COMPAS for each year and for each of the three cost items considered (outpatient consultations with generalists and specialists are projected and adjusted separately, but reported jointly). These calculations, done using a 3% real discount rate, show that the present value of these health cost savings until 2050 amounts to C$13.1 billion (10.8; 15.7), in 2012 C$, under the incidence-based scenario (Table 3). In contrast and as expected, the mortality-based scenario (Table 4) yields negative savings—hence additional costs—of C$3.4 billion (-5.4; -1.4), in 2012 C$. All these results are robust to model uncertainty.

**Table 3:** Total discounted value of savings in 2012 for a reduction in CVD incidence to decrease mortality by 25% by 2025 “incidence-based” scenario (billions of 2012C$).

	Horizon 2020	Horizon 2035	Horizon 2050
Hospitalizations	0.42 (-0.33; 1.24)	4.36 (3.16; 5.76)	9.82 (7.97; 11.90)
Consultations with specialists	0.11 (-0.07; 0.35)	0.93 (0.48; 1.31)	2.17 (1.53; 2.74)
Consultations with generalists	0.05 (0.01; 0.12)	0.49 (0.40; 0.59)	1.15 (0.96; 1.32)
**Sub-Total: Healthcare**	**0.58 (-0.24; 1.51)**	**5.78 (4.40; 7.61)**	**13.10 (10.80; 15.70)**
Life expectancy at age 30	0.71 (-4.73; 7.34)	12.20 (-4.36; 27.90)	38.20 (11.90; 65.00)
**Total**	**1.30 (-4.17; 7.34)**	**17.90 (2.20; 33.70)**	**51.40 (25.10; 77.10)**

Notes: 3% discount rate used. Each column shows the present discounted value for a given horizon (e.g. horizon 2020 refers to the present value of the flows from 2012 to 2020). Each life-year saved is valued at C$200,000. Confidence intervals at the 5^th^ and 95^th^ percentiles are shown in brackets for each line.

**Table 4:** Total discounted value of savings in 2012 for a 25% reduction in CVD mortality by 2025: “mortality-based” scenario (billions of 2012C$).

	Horizon 2020	Horizon 2035	Horizon 2050
Hospitalizations	-0.08 (-0.91; 0.73)	-0.93 (-2.28; 0.40)	-2.48 (-4.27; -0.91)
Consultations with specialists	0.01 (-0.22; 0.22)	-0.21 (-0.59; 0.11)	-0.55 (-1.18; -0.02)
Consultations with generalists	0.00 (-0.05; 0.04)	-0.14 (-0.24; -0.04)	-0.37 (-0.53; -0.19)
**Sub-Total: Healthcare**	**-0.07 (-1.01; 0.77)**	**-1.27 (-2.51; 0.06)**	**-3.40 (-5.40; -1.46)**
Life expectancy at age 30	1.66 (-4.99; 7.70)	28.70 (10.30; 45.90)	69.60 (41.20; 96.60)
**Total**	**1.59 (-5.38; 7.67)**	**27.40 (9.88; 44.70)**	**66.20 (38.70; 91.80)**

Notes: 3% discount rate used. Each column shows the present discounted value for a given horizon (e.g. horizon 2020 refers to the present value of the flows from 2012 to 2020). Each life-year saved is valued at C$200,000. Confidence intervals at the 5^th^ and 95^th^ percentiles are shown in brackets for each line. “CVD Mortality” refers to all-cause mortality for individuals with a self-reported diagnosis of heart disease or stroke in the previous 2-year cycle.

We can add to these cost savings the value associated with longevity extension, in monetary units to provide a common metric with healthcare cost savings. After computing the number of “years of life saved” aggregated for all of Quebec in each year, as reported above in Table 2, we use a value of $200,000 for the statistical value of a life year, a figure often used in the literature [12]. We also use a real discount rate of 3% for this component. As shown in Tables 3 and 4, we estimate the value of all the years of life saved from 2012 to 2050 to be C$38.2 billion (11.9; 65.0) or C$69.6 billion (41.2; 96.6), depending on the scenario—but in all cases far exceeding the value of healthcare costs savings, if any. These results are robust to model uncertainty, since 98% (in the incidence-based scenario) or 100% of our model replications (in the mortality-based one) yield positive benefits.

## Discussion

Combined, the flows to 2050 of healthcare cost savings and of the value of years of life saved with standard assumptions under the mortality-based scenario, in which death rates of individuals suffering from CVD decrease directly, are worth over C$66 billion in 2012 dollars—a significant figure for Quebec, whose gross domestic product is under C$400 billion. This value stems only from the value of life years, however, since healthcare costs in major categories (hospitals and physicians) are projected to increase faster in this scenario than under the baseline, potentially making any intervention towards it a hard sell for the healthcare payer (in Quebec, the provincial government).

On the other hand, the incidence-based scenario yields more modest gains in life expectancy that take more time to materialize, leading to a lower value of this component of the benefits. The substantial flow of healthcare cost savings partly counterbalances this, such that the value of the benefits of the reduced CVD incidence still totals over C$51 billion in 2012 dollars.

### Sensitivity analyses

**Fig 3:**
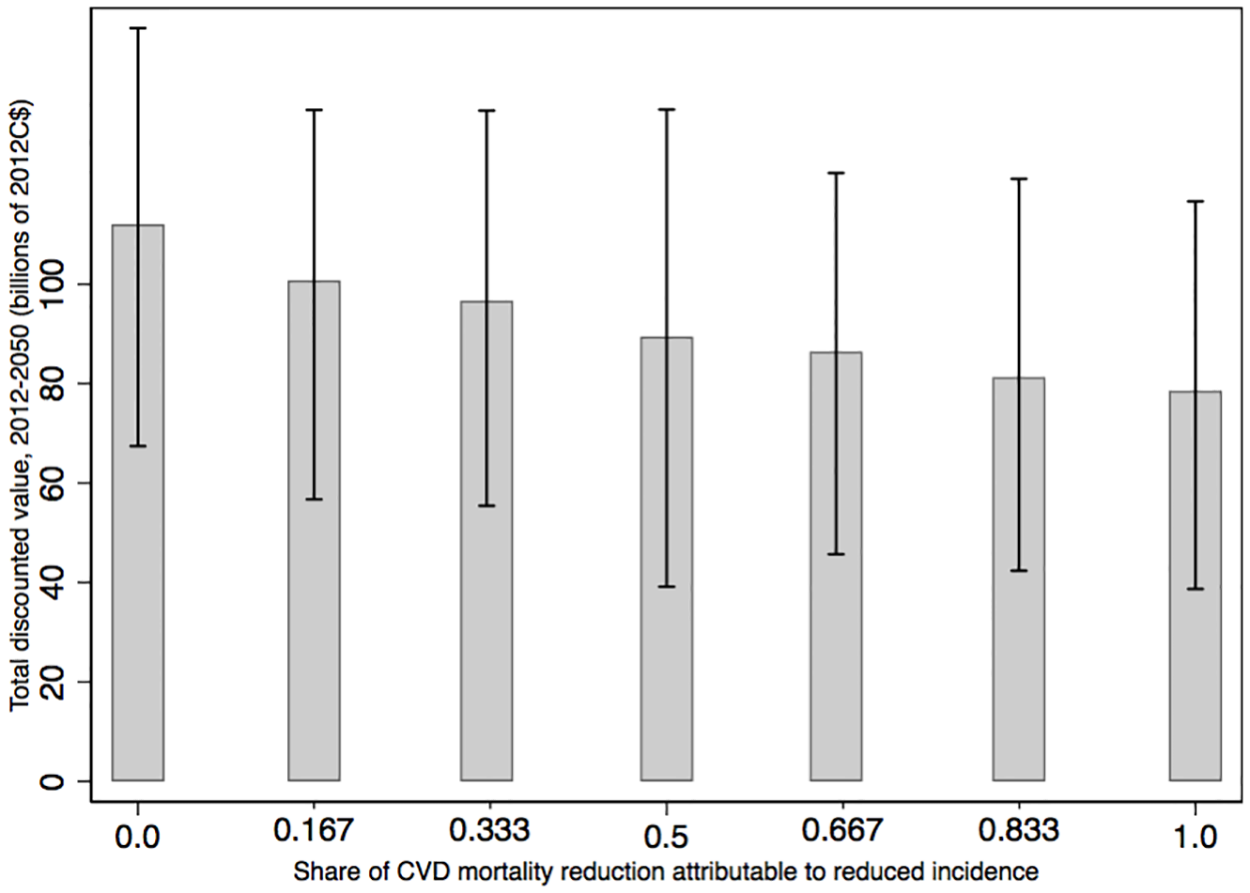
Total discounted value of healthcare cost savings and years of life saved for combinations of the incidence and mortality scenarios, 2012 to 2050 (billions of 2012C$). Notes: The share of CVD mortality reduction by 2025 achieved through lower CVD incidence (“prevention”), as opposed to lower mortality among individuals suffering from CVD (“treatment”), is varied from zero to one. Confidence intervals at the 5^th^ and 95^th^ percentiles are shown.

Other than uncertainty in the estimates, we carried out two further sensitivity analyses in addition to our simulated scenarios and to the previously mentioned sources of uncertainty inherent to COMPAS. First, we model a combination of pathways to achieving the WHO target of a 25% reduction in CVD mortality by 2025. We varied from 0 to 1 the share of CVD mortality reduction by 2025 achieved through lower CVD incidence (“prevention”), as opposed to lower mortality among individuals suffering from CVD (“treatment”)–always accounting for the dynamic relationship between CVD incidence and associated mortality. This creates combinations of our two main scenarios, which provides an illustration of how total benefits of target achievement vary—they do vary because benefit flows differ between the two main scenarios. Fig 3 illustrates the results, which provide an indication of the benefits of combinations of potential interventions to achieve the WHO target. Although a higher a proportion of reduced CVD mortality being achieved directly is associated with a larger discounted value, all average values stand inside the 90% confidence intervals of all other combinations, which is an indication that they may not actually differ much from one another.

The discount rate used in computing the value of the future flows of healthcare costs and years of life has also been varied to test the sensitivity of our results: real discount rates of 1% and 5% were tested in addition to the baseline 3%. Because our outcomes of interest are measured over a long period, discounting can make an important difference, with a lower (higher) discount rate yielding larger (smaller) benefits—and this is the case here, given that an important share of the benefits in our scenarios occur in the future.

The value of life expectancy gains in the incidence-based scenario thus nearly doubles when using a 1% discount rate, to over C$65 billion. The mortality-based scenario yields a staggering C$115 billion in value for life-years saved. The same holds for healthcare costs, with the savings amplified to C$21.4 billion in the incidence scenario and the additional costs totaling C$5.7 billion in the mortality-based scenario. Mechanically, a 5% discount rate has the opposite effect; using a 5% rate cuts all values by approximately 1/3 relative to using a 3% rate. In all cases, regardless of the discount rate used, the incidence-based scenario generates significant savings in terms of healthcare costs and life years, while the mortality-based scenario yields additional healthcare costs (negative savings) but tremendous benefits in terms of years of life saved.

### Strengths and limitations

COMPAS generates with great detail the probability of every individual going through a health transition based on his or her personal characteristics and at various stages in life. For each individual, interactions between CVD and other major diseases are accounted for in the model. Hence, estimates that include dynamic effects may differ from static estimates. However, it is unclear whether such estimates are lower or higher than static estimates because of two competing effects: since those with a condition may die earlier, the direct lifetime burden may be lower using a dynamic approach. On the other hand, the dynamic approach delivers feedback effects from preventing one disease to another, which may deliver larger lifetime effects. Our projections of healthcare use and related costs do present an important limitation: we do not account for direct effects on productivity and labor force participation. We also do not account for changes in drug spending since the administrative data we used did not allow to track medication consumption. Finally, the benefits associated with an improved life expectancy will likely keep accruing for years beyond our projection period. Hence, our estimates are probably a lower bound on the aggregate economic effect of a reduced CVD mortality.

## Conclusions

Over the past 60 years, remarkable progress has been made in combating CVD in Canada. Mortality rates have declined in significant ways, and this is attributable to a combination of advances in risk factor reduction and CVD treatment and management [22]. Yet, heart disease and stroke remain the leading causes of death and hospitalization and the biggest driver of prescription drug use in Canada.

A greater understanding of healthcare use patterns and the related costs may help improve the targeting of patients, where a number of key management decisions may help contain costs. This study shows that without technological progress or changes in lifestyle habits, government spending for healthcare in Quebec will significantly increase. Between 2012 and 2050, the costs of consultations with physicians and the costs of hospitalizations are projected to increase by slightly more and slightly less than 100%, respectively, leaving aside inflation.

The scenario of a 28% reduction in CVD incidence by 2025, aimed at reducing age-adjusted death rates by 25% for those who suffer from CVD, could contain the pace of healthcare cost increases by nearly 9 percentage points for hospitalizations and over 6 percentage points for consultations with a physician. Combined, these savings would represent over C$1.1 billion in healthcare use for 2012, or 4% of the Quebec government’s healthcare expenditures that year [23]. The mortality-based scenario, on the other hand, would entail higher healthcare costs.

Over the entire period to 2050, the flow of cost savings in the incidence-based scenario, perhaps more oriented toward primary prevention, is projected to amount to C$13.1 billion in 2012 dollars. The dollar value of years of life saved due to improved life expectancy with a reduction in CVD achieved through lower incidence could be worth an additional C$38.2 billion. This is less than the value of addressing CVD mortality directly which, despite the higher health costs that would result, could save years of life worth C$69.6 billion—in part because life expectancy would improve more quickly than under the incidence-based scenario. These estimates do not account for the direct costs of interventions that would have to be implemented to achieve this CVD reduction target. Nevertheless, even though our two scenarios—even the one generating monetary savings—would not solve Canada’s healthcare financing challenges, potential savings are considerable; so much so that they certainly call for more prevention to reduce CVD, including efforts to tackle diabetes and hypercholesterolemia. They would also yield tremendous value for Quebec in terms of years of life saved at the population level, as well as of their value expressed in monetary units.

## Supporting information

S1 File The economic benefits of reducing cardiovascular disease mortality in Quebec, Canada: Technical Appendix. This technical appendix provides further information on the COMPAS model, including on the topics noted in the article.
(PDF)
