# Supplementary material for: The economic benefits of reducing cardiovascular disease mortality in Quebec, Canada
Source: PLoS One. 2018 Jan 4;13(1):e0190538. doi: 10.1371/journal.pone.0190538 (PMC5754059; doi:10.1371/journal.pone.0190538)
Supplement: S1 File — This technical appendix provides further information on the COMPAS model, including on the topics noted in the article. (PDF) [file pone.0190538.s001.pdf]

The economic benefits of reducing cardiovascular disease mortality in Quebec, Canada[1]

Technical Appendix

David Boisclair, HEC Montréal
Yann Décarie, Institut national de la recherche scientifique (INRS) - Urbanisation, culture et société
François Laliberté-Auger, HEC Montréal
Pierre-Carl Michaud, HEC Montréal, CIRANO, RAND[2]
Carole Vincent, Consultant in Social and Economic Studies

December 2017

[1]We wish to thank the following current and former collaborators for their contribution to the development of COMPAS: Aurélie Côté-Sergent, Jean-Yves Duclos, Guy Lacroix, Alexandre Lekina, and Steeve Marchand.
[2]Contact: Département déconomie appliquée, HEC Montréal, 3000, chemin de la Côte-Sainte-Catherine, Montréal (Québec), H3T 2A7, Canada.

Contents

1 COMPAS: Context and model overview **1**
1.1 Context . . . 1
1.2 Dynamics of COMPAS . . . 1

2 Main survey data used **4**
2.1 National Population Health Survey (NPHS): Description . . . 4
2.2 Construction of the health variables using the NPHS . . . 7
2.3 Canadian Community Health Survey: Description and construction of the variables . . 12

3 Initial population **14**
3.1 Imputation of variables and calibration . . . 14
3.2 Characteristics of the initial population . . . 15

4 Health status transitions **17**
4.1 Econometric models . . . 17
4.2 Transitions validation . . . 24

5 Population renewal **25**

6 Demography: Mortality and immigration **26**
6.1 Mortality . . . 26
6.2 Immigration . . . 28
6.3 Conclusion . . . 28

7 Health care use **30**
7.1 Modelling . . . 30
7.2 Results . . . 32
7.3 Conclusion . . . 34

8 Health care costs **35**
8.1 Hospital stays: MED-ECHO data and NIRRU . . . 35
8.2 Physicians: RAMQ data . . . 36
8.3 Models . . . 36
8.4 Aggregate costs . . . 39

9 Uncertainty **40**
9.1 Types of uncertainty 40
9.2 Presentation of the uncertainty 40
9.3 Summary 41

Bibliography **43**

List of Tables

2.1 Number of observations for each Canadian region and for Canada in the NPHS (1994-2011) 5
2.2 Annual mortality rate by NPHS cycle, population aged 30 and over 6
2.3 Dimensions of health status accounted for in COMPAS 8
2.4 Dimensions of health care use accounted for in COMPAS 8
2.5 Self-reported disease prevalence in the NPHS (1994-2011) 9
2.6 Self-reported disease incidence in the NPHS (1994-2011) 9
2.7 Self-reported disease remission in NPHS (1994-2011) 10
2.8 Health care use by age according to the NPHS (1994-2011) 11
2.9 Self-reported disease prevalence in the CCHS (2008-2009 and 2010 combined) 13

3.1 Description of the initial population (mean of 100 replications) 16

4.1 Permitted effects of diseases (rows) on other diseases' incidence (columns) 18
4.2 Average marginal effects of variables on the probabilities of disease incidence over two years 20
4.3 Average marginal effects of variables on the probabilities of disease remission 21
4.4 Average marginal effects on two-year probability of death 22
4.5 Difference in prevalences between simulated population and NPHS population — 1994-2010 24
4.6 Comparison of projected life expectancies for the province of Quebec 24

6.1 Annual rates (in %) of mortality rates reduction. 27
6.2 Comparison of projected population for Canada: COMPAS vs. Statistics Canada . . 29

7.1 Average marginal effect of different variables on health care use. 33

8.1 Average marginal effect of the different variables on the annual cost of hospitalization . 37
8.2 Average marginal effect of each variable on the annual cost of consultations 38
8.3 Aggregate expenses on hospitals and physicians in 2012 according to CIHI/RAMQ and COMPAS, and scaling ratios 39

Chapter 1

COMPAS: Context and model overview

1.1 Context

This technical appendix is a streamlined version of the full technical documentation that can be found in the GitHub archive[1]

1.2 Dynamics of COMPAS

COMPAS has two important components: 1) a dynamic component that allows us to simulate individuals progressing through their life cycle, and thus also to incorporate the evolution of their health status, captured by various indicators such as the presence of illnesses, the presence of activity limitations, etc.; and 2) a cross-sectional component aiming to quantify the cost of medical resource use associated with the health status of persons alive in a given year. The second component will benefit from the rich infrastructure of existing data, including – for Quebec – administrative data from the Régie de l'assurance maladie du Québec (RAMQ) as well as from the Ministry of Health and Social Services.

The model begins in 2010, the most recent year for which reliable data is available for some (crucial) sources of information that we use. For that year, we create an initial population using an existing survey. This initialization phase gives a statistically representative sample of Canada – as well as Quebec, Ontario and other provinces grouped into regions – in 2010. Then begins the process of demographic evolution of the population up to the target year: 2050. Depending on the desired output variables, the model can keep estimating its variables up to the year 2130 if any remaining agents are still alive. This could be useful, for example, to analyze the evolution of life expectancy of each cohort entering the model. In effect, cohorts entering the model between 2010 and 2050 will see a

[1] Full technical documentation : https://github.com/CEDIA-models/compascvd2017/tree/master/documentations/Full Technical Documentation.pdf.

major proportion of agents who remain living in 2050 (the oldest will only be 70 years old). Figure 1.1 summarizes how the model works and the dynamics of COMPAS.

The microsimulation model includes several components. The structure of the model is almost entirely based on the population dynamics. The model tracks agents, characterized by socioeconomic and health attributes, which transition from one period to another. After the initial year, new agents enter the simulation at the beginning of each simulation cycle[2] at the default starting age(s), which we set at 30-31 years old, or at any age if they immigrate.[3] Their life in the model ends at their time of death or when they reach the maximum age permitted in the model, which we have set at 110 years old.

Each period, the living population is characterized by a set of demographic, economic and health variables. The health status variables make it possible to attribute to individuals a level of health care use and related expenses in a given period. This population then passes through a transition phase where health status may change. For example, an individual aged 80 years with hypertension would have a set of probabilities of suffering from a stroke. These probabilities depend on his current health status. During this transition, each individual faces a risk of dying or of developing one or more disabilities which, for instance, increase their probability of entering a long-term care facility in subsequent cycles.

In each simulation cycle, we can observe not only individuals' health status, but also their use of health care. For the purpose of running this cross-sectional component, the population includes agents who are alive at the end of a given period. In each simulation cycle, the population moves forward in time. It loses some agents due to mortality, and gains some because there is renewal – i.e., a new cohort of agents aged 30-31 years old. enters the model. Finally, the population gains and loses members due to migration. Each simulated agent has a given demographic weight; thus, statistics of interest can be computed at the population level. Since the simulations include a stochastic element, the model enables the user to make several replications in order to ensure that the result obtained is not purely a matter of chance.

[2] In COMPAS, a simulation cycle corresponds by default to a period of two years.

[3] Age of entry is set at 30-31 years old in order to avoid having to model individual education trajectories and choices, and because illnesses and disabilities considered in the model are uncommon before this age.

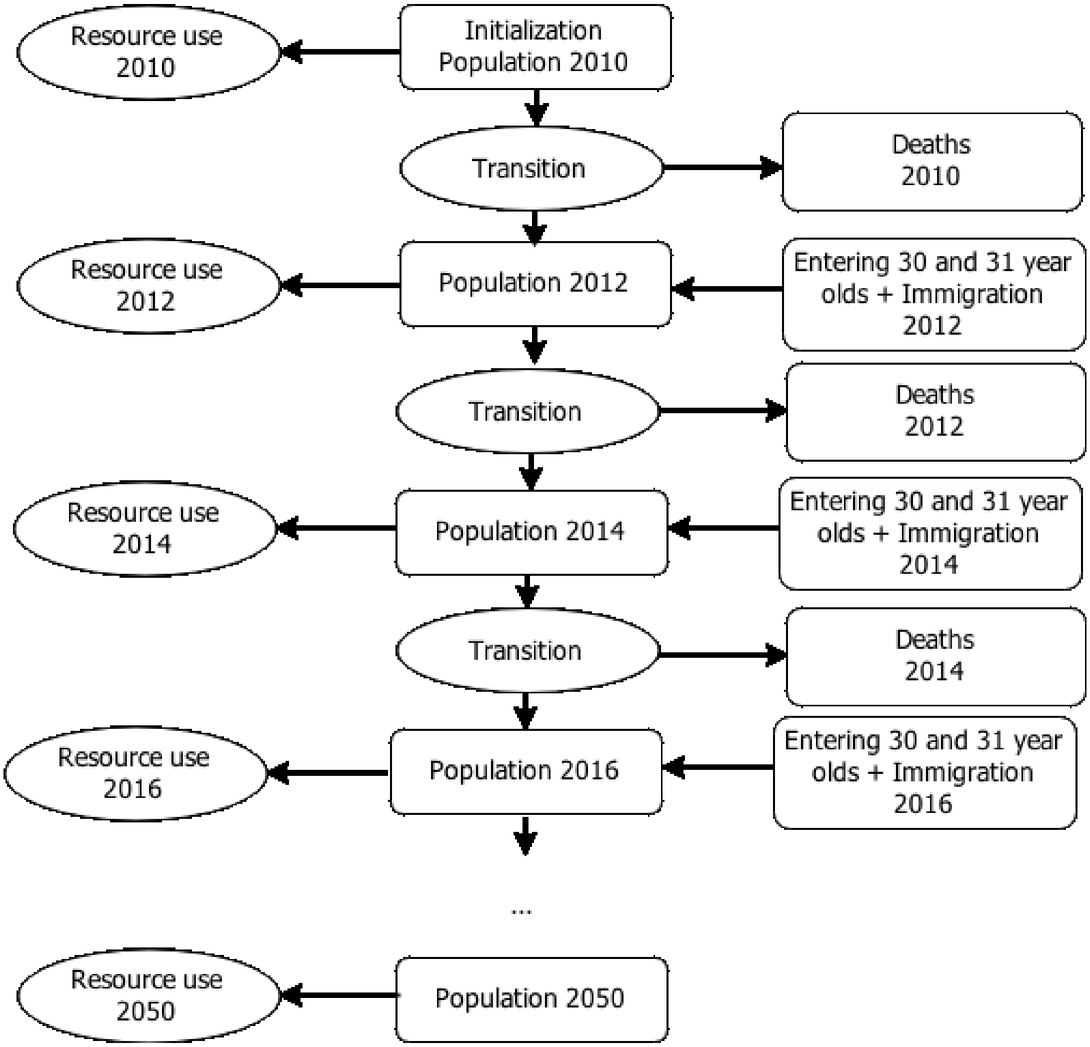

Figure 1.1: Dynamics of the COMPAS model with 2-year simulation cycles

Chapter 2

Main survey data used

This chapter explains how the data used to build the microsimulation model is prepared. We use the National Population Health Survey (NPHS), the only longitudinal survey on health in Canada, to build the transitions module and the health care use module. The 2008-2009 and the 2010 Canadian Community Health Survey (CCHS) are used to construct the model's initial population. These two surveys are representative of the Canadian population around 2010. For the operation of the model, we minimally need these three surveys, the use of which is described in the following sections. For convenience and because some of them are otherwise difficult to locate, the questionnaires of these surveys are made available in our GitHub archive (https://github.com/CEDIA-models/compascvd2017).

In addition, we use the General Social Survey (GSS) to estimate some health care use. We also use the Labour Force Survey (LFS) to estimate trends and to impute certain levels of education. We also use more data provided by Statistics Canada regarding population size by age to mimic its distribution in the model's initial population. Finally, we use data from the Régie de l'assurance maladie du Québec (RAMQ) to calculate some health care costs. More details regarding these complementary sources of data are provided in subsequent chapters.

2.1 National Population Health Survey (NPHS): Description

The NPHS is a longitudinal survey carried out on a sample of the Canadian population. The questionnaire was administered on the same sample of respondents every two years from 1994-1995 onwards. There are nine cycles in this survey, the last of which being 2010-2011. While there are three components to the NPHS (the Households component, the Health Institutions component, and the North component), only the Households component is used in this model. The North component has not been implemented by the NPHS since 2000-2001, while the Health Institutions component was terminated in 2002-2003.

In the Households component, all respondents interviewed necessarily live in a private household in the first cycle of the survey. The survey thus excludes individuals residing on Indian reserves, on Crown land and in remote regions of Quebec and Ontario, as well as full-time members of the Canadian Forces and persons living in institutions (Statistics Canada, 2012a). It should be noted that if a respondent

from the Households component moves to a long-term care institution between two cycles of the survey, she is still followed in the Households component.

In 1994, there were 12,797 respondents aged 30 and over in Canada, including 3,143 aged 65 years old or over. In comparison, there were 1,866 (3,838) respondents in Quebec (in Ontario), including 365 (984) aged 65 years or over. This comparison suggests that the number of observations in each region is small, especially for the population aged 65 and over. Given that certain health problems are relatively rare, it seems prudent to use the entire Canadian sample and to allow deviations for each region considered in the model. These regions, five in total, are the Atlantic Provinces (Nova Scotia, New Brunswick, Prince Edward Island and Newfoundland), Quebec, Ontario, the Prairies provinces (Manitoba, Saskatchewan and Alberta), and British Columbia. Table 2.1 presents the number of observations in the NPHS by age group for each region.

	Atlantic Provinces	Quebec	Ontario	Prairies	British Columbia	Canada
30 to 34 years old	396	283	594	410	251	1,934
35 to 39 years old	360	285	515	369	241	1,770
40 to 44 years old	314	252	435	288	210	1,499
45 to 49 years old	315	214	395	284	184	1,392
50 to 54 years old	243	180	318	218	150	1,109
55 to 59 years old	221	141	311	202	132	1,007
60 to 64 years old	202	147	286	192	116	943
65 to 69 years old	210	131	304	201	115	961
70 to 74 years old	204	116	307	199	83	909
75 to 79 years old	147	65	190	163	73	638
80 years old and older	154	52	183	165	81	635
Total (30 years and older)	2,766	1,866	3,838	2,691	1,636	12,797

Table 2.1: Number of observations for each Canadian region and for Canada in the NPHS (1994-2011)

2.1.1 Weighting

Appropriate weights are built by Statistics Canada and used in COMPAS. Information on weights in the NPHS can be found in section 2.1.1 of the full technical documentation.

2.1.2 Attrition

Attrition is always a concern in longitudinal surveys; in the NPHS it appears to be of a magnitude similar to that found in other surveys. A complete discussion of attrition in the NPHS can be found in section 2.1.2 of the full technical documentation.

2.1.3 Mortality

The NPHS provides two ways of detecting mortality. The first is for an interviewer to obtain the information when attempting to contact the respondent. The second is by validation with the national

registry of deaths. Such a validation is only possible when respondents had given permission for their survey data to be linked to administrative data. About 90% of respondents had given permission for data linkage.

Table 2.2 presents the annual mortality rates of the NPHS by cycle for the population aged 30 years and over. In earlier cycles, the rates increase over time. In the later cycles, the annuel mortality rate decreases. We believe this is mainly due to problems with the validation from the national death registry. Indeed, the validation with the registry is performed retrospectively for the previous cycle. As we have seen, there are nine cycles in the NPHS; but no deaths have been validated with the registry in the last cycle (2010-2011), which looked at deaths that occurred among individuals surveyed in the previous cycle.[1] We therefore exclude the eighth wave (2008-2009) for mortality estimation purposes, since death records are not complete for these individuals. Furthermore, no information on deaths is available after the last cycle of the survey (i.e. for the individuals surveyed in 2010-2011).

	Mortality rates
1994-1995	0.98%
1996-1997	1.13%
1998-1999	1.13%
2000-2001	1.15%
2002-2003	1.28%
2004-2005	1.25%
2006-2007	1.05%
2008-2009	0.85%
2010-2011	-
1994-2010	1.10%

Table 2.2: Annual mortality rate by NPHS cycle, population aged 30 and over

Figure 2.1 presents the mortality rates by age from the NPHS from 1994 to 2006, as well as the 95% confidence interval around these rates (dotted lines). We compare these rates with the periodic mortality rates from the Human Mortality Database for a similar period, i.e. from 1995 to 2004. All Canadian data comes from Statistics Canada.

The fit is satisfactory up to about 75 years of age. However, there is divergence after 75 years, that is from the moment where the number of individuals in institutions strongly increases. We cannot fully explain this phenomenon at the moment, but we discuss in the next chapter how mortality rates are calibrated in the first year of the simulation to take these differences into account.

[1] This is shown in a chart in the NPHS's User Guide, only available in Statistics Canada's Research Data Centres (RDCs).

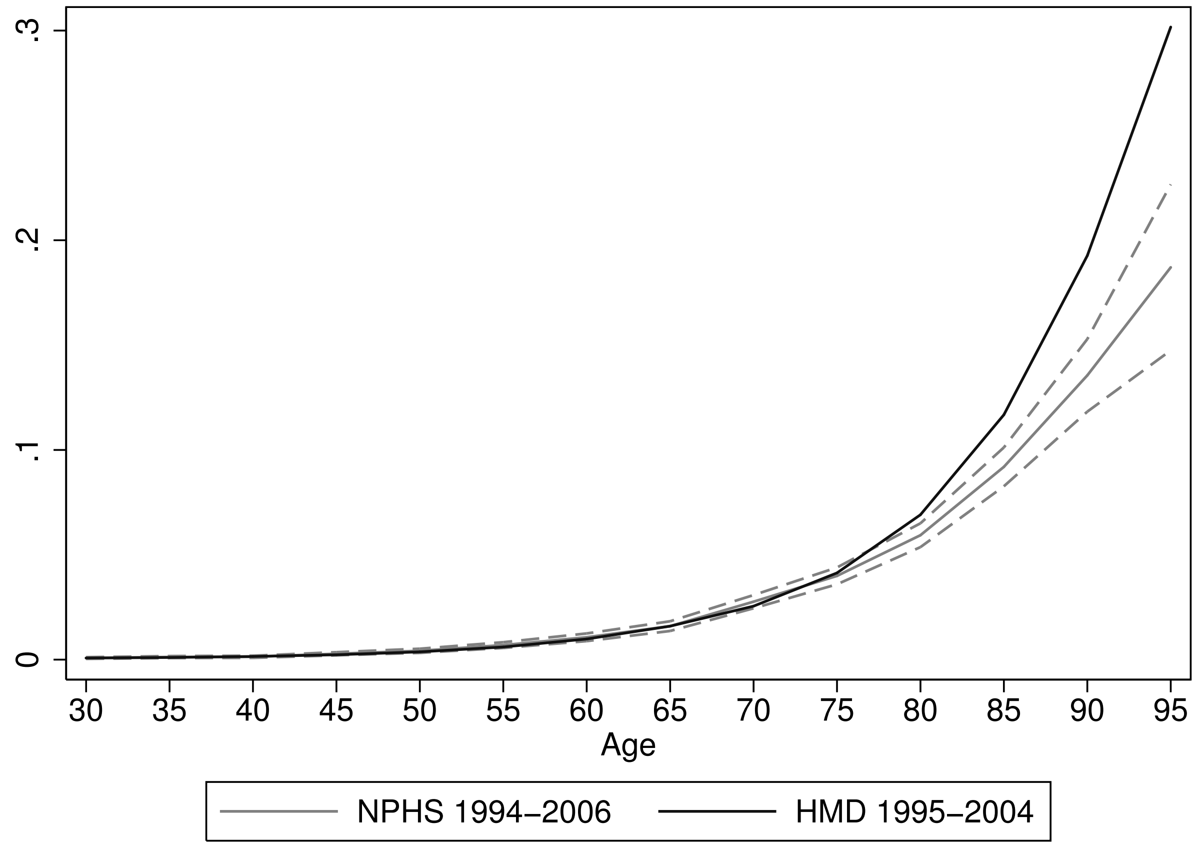

Figure 2.1: Comparison of mortality rates: NPHS vs. Human Mortality Database (HMD).
Note: dotted lines show the 95% confidence interval.

2.2 Construction of the health variables using the NPHS

The NPHS questionnaire is rich, and the questions are for the most part identical from one cycle to the next. A person's health status includes many dimensions in the model; the dimensions considered are presented in Table 2.3. For health care use, COMPAS accounts for the dimensions shown in Table 2.4, but this article only considers the first three – physician visits and nights spent in hospital.

We use the sampling weights to produce the statistics presented hereafter. In this version, we have not stratified by province nor by gender. However, in order to check the existence of differences between certain regions and the country as a whole, we present for all results Student's comparison tests, which indicate whether the proportion for Canada is significantly different from the proportion for each region.[2]

[2]Throughout the tables in this chapter, a negative (-) t-statistic indicates a lower proportion in the designated region compared to Canada as a whole, while a positive t-statistic indicates a higher proportion in the region. The significance of these differences depends on the size of the statistics; e.g., an absolute value of more than 1.96 indicates a significant difference at a 5% threshold. The direction of the difference depends on the sign of the t-statistic, as explained.

Diabetes
Hypertension
Cancer
Stroke
Heart disease
Lung disease
Alzheimer's and other dementias
Obesity (BMI)
Tobacco use
Presence of disabilities

Table 2.3: Dimensions of health status accounted for in COMPAS

Number of visits to a generalist
Number of visits to a specialist
Number of nights of short-term hospitalization
Use of medicines (yes / no)
Use of home care services (yes / no)
Use of home care services (formal / informal / both)

Table 2.4: Dimensions of health care use accounted for in COMPAS

2.2.1 Self-reported health conditions

The model covers seven self-reported health conditions: presence of diabetes, of hypertension, of cancer, of heart disease, of strokes, of lung disease, and of dementias (Alzheimer's and others). These diseases were selected primarily for their high prevalence in the population and their presence in others micro-simulation models like the *Future Elderly Model* (Goldman et al., 2005), allowing for comparisons.

The questions are asked as follows in the NPHS: *"We're interested in conditions diagnosed by a health professional [...]. Do you have diabetes?, ... hypertension?, etc."* (Statistics Canada, 2012b). We consider the presence of diabetes, hypertension, lung disease or dementia to be an absorbing state. We therefore recode these variables such that after a first positive response, individuals are considered to have the condition in all the following cycles. For the three others health conditions (cancer, heart disease and stroke), we model remissions, and as such we keep the data as is. Once aggregated, the answers of all respondents lead directly to measures of prevalence. To develop the measures of incidence, we use positive responses in the current cycle when the response was negative in the preceding cycle – accounting for the absorbing states defined as mentioned above.

Interpreting the prevalence by age is difficult for two important reasons: a) cohort effects (intergenerational differences for a given age) in disease incidence; and b) increased mortality among individuals suffering from a disease. Thus, we could very well observe that incidence increases with age while prevalence decreases. We present both prevalence and incidence for each disease.

Table 2.5 gives the prevalence rates by 10-year age group for each disease. Unsurprisingly, we find that prevalence increases with age, except for certain diseases (diabetes, cancer) for which prevalence decreases after 90 years. Higher mortality among individuals with those conditions may explain this pattern. The most common disease among individuals aged 40 years and older is hypertension, by a fairly wide margin – though heart disease prevalence increases substantially at older ages.

Age	Diabetes	Hypertension	Cancer	Heart disease	Stroke	Lung disease	Dementias
30 to 39 years	1.4%	5.6%	0.4%	0.9%	0.2%	6.1%	0.3%
40 to 49 years	3.6%	11.0%	0.8%	1.9%	0.3%	6.5%	0.3%
50 to 59 years	7.4%	26.3%	2.1%	5.0%	0.9%	7.2%	0.4%
60 to 69 years	12.7%	42.4%	3.6%	10.5%	2.2%	9.7%	1.1%
70 to 79 years	17.9%	52.0%	4.5%	17.6%	4.41%	13.1%	3.3%
80 to 89 years	19.6%	58.0%	6.4%	24.9%	7.5%	16.2%	9.9%
90 years and older	18.0%	57.3%	7.0%	22.8%	10.6%	17.2%	17.7%
Total (30 yrs +)	7.6%	24.9%	2.1%	6.5%	1.5%	8.3%	1.3%
t-statistics of difference between rates in a given region and those in Canada							
Atlantic Provinces	-5.17	-9.45	-1.79	-0.79	1.10	-2.89	0.92
Quebec	1.33	2.14	4.61	2.80	0.81	0.31	0.15
Ontario	-1.59	-2.30	-2.27	-5.05	-1.44	-2.48	2.59
Prairies	2.67	4.08	-0.71	7.04	0.04	5.45	-4.51
British Columbia	3.22	3.80	-0.55	3.26	3.32	2.05	1.41

Table 2.5: Self-reported disease prevalence in the NPHS (1994-2011)

In Table 2.6, we present the incidence rates by age group and for each disease. Among those aged 40-49 years, incidence is highest for hypertension, with 2.3%. Indeed, hypertension exhibits the highest incidence up until 70-79 years. We also note that incidence generally increases with age, although it declines for some diseases at advanced ages. A number of reasons could explain that, including selection and the biological processes leading to these diseases.

Age	Diabetes	Hypertension	Cancer	Heart disease	Stroke	Lung disease	Dementias
30 to 39 years	0.35%	1.15%	0.31%	0.75%	0.03%	0.59%	0.02%
40 to 49 years	0.64%	2.30%	0.65%	1.22%	0.20%	0.54%	0.06%
50 to 59 years	1.36%	4.97%	1.42%	2.55%	0.40%	0.83%	0.10%
60 to 69 years	1.76%	5.69%	2.32%	4.87%	1.01%	1.28%	0.34%
70 to 79 years	2.16%	5.58%	3.01%	8.31%	2.12%	1.54%	1.53%
80 to 89 years	1.77%	4.89%	4.08%	12.15%	3.36%	2.10%	5.19%
90 years and older	1.20%	5.28%	4.55%	13.26%	7.91%	4.80%	8.67%
Total (30 yrs +)	1.05%	3.32%	1.35%	2.93%	0.75%	0.88%	0.53%
t-statistics of difference between rates in a given region and those in Canada							
Atlantic Provinces	-2.47	-0.76	-0.93	-0.76	-0.20	-1.07	1.96
Quebec	0.07	-0.87	0.59	-0.64	0.51	-1.59	-0.08
Ontario	-0.31	-0.18	-0.27	-1.00	-0.20	0.73	0.32
Prairies	1.55	0.74	-0.46	2.24	-0.11	0.04	-1.57
British Columbia	0.91	1.86	1.29	1.97	0.44	2.00	1.06

Table 2.6: Self-reported disease incidence in the NPHS (1994-2011)

In Table 2.7, we present the remission rates by age group for cancer, heart disease and stroke, the three conditions for which remissions are allowed. The percentage of remissions for cancer decreases sharply with age, from 70.6% for individuals between 30 and 39 years old to 37.6% for people 90 years and over. We do not observe the same pattern for heart disease, where remissions vary between 25.6% and

30.9% – except for the 30-39 years old and the 90 years and over, who experience a higher remission rate. Remission from suffering from the effect of a stroke varies between 16.7% and 24.8%, with the exception of the oldest age group in which we observe the highest rate of remissions at 33.3%.

	Cancer	Heart disease	Stroke
30 to 39 years	70.62%	45.07%	19.68%
40 to 49 years	57.87%	30.51%	20.36%
50 to 59 years	56.38%	25.61%	16.73%
60 to 69 year	54.12%	30.94%	19.34%
70 to 79 years	53.82%	26.75%	23.16%
80 to 89 years	48.61%	29.97%	24.77%
90 years and older	37.59%	49.21%	33.29%
Total (30 yrs +)	54.86%	29.56%	21.77%

Table 2.7: Self-reported disease remission in NPHS (1994-2011)

2.2.2 Disability

We sought to build variables that could take into account cognitive and physical disabilities. Thus, we use the variables from the NPHS questionnaire to build one measure of cognitive impairment and two measures of physical disability.

Section 2.2.2 of the full technical documentation more information on disability in COMPAS.

2.2.3 Risk factors

COMPAS currently accounts for two major risk factors, i.e. obesity and smoking. We have defined three smoking statuses: 1) never smoked; 2) current smoker; and 3) former smoker. As for obesity, we have constructed a variable based on the body mass index (BMI), with three categories (under 30, 30-35, and 35+).

More information on risk factors can be found in section 2.2.3 of the full technical documentation.

2.2.4 Health care use

The NPHS is rich in data on health care use (Statistics Canada, 2012b). Six variables were created for modelling purposes. These were chosen first because the information was available in the NPHS. Secondly, they represent an important part of health expenditures. The first two variables are the number of visits or telephone consultations over the previous 12 months, on the one hand with a family doctor, paediatrician or general practitioner; and on the other hand with another doctor or specialist (e.g. a surgeon, an allergist, an orthopedist, a gynecologist or a psychiatrist). These two variables were censored at the 99th percentile because the last observations presented a high number of consultations, which might skew the means.

Since doctor consultations do not include stays in a care facility, a third variable was created: the number of nights spent in a hospital, a non-residential nursing home or a convalescent home. As was the case for medical consultations variables and for the same reason, this variable was censored at the 99th percentile. This third category does not include same-day surgeries and emergency room visits, since these are excluded from the NPHS. They are therefore excluded from COMPAS for now.

The fourth variable is a binary variable indicating whether the individual took at least one medication (prescription or over-the-counter) in the previous month. The fifth variable takes three possible values, indicating whether the individual 1) is not receiving long-term care; 2) is receiving home care; or 3) resides in a long-term care facility. The sixth variable also takes three possibles values, indicating whether the home care received was 1) formal; 2) informal; or 3) both formal and informal. More information on the three latter variables, which are not used for this article, can be found in chapter 2 of the full technical documentation.

Table 2.8 gives the distribution by age for the first three variables. The number of consultations with a general practitioner increases fairly constantly with age, especially beyond 50 years (one may suppose that, between the ages of 30 and 39 years, pregnancy involves a higher number of visits). Over the course of the 12-month reference period, individuals consult a general practitioner three times on average. Consultations with specialists in the previous 12 months, however, drop to under one. The number of nights of hospitalization increases faster after 70 years of age. Individuals aged 80 to 89 years spend on average more than three nights in a short-term facility over the previous 12 months.

Health care use by disease status is presented in section 2.2.4 of the full technical documentation.

Age	Nb consultations w/ generalist	Nb consultations w/ specialist	Nb hospitalization nights
30 to 39 years	2.69	0.70	0.46
40 to 49 years	2.57	0.66	0.45
50 to 59 years	3.01	0.78	0.70
60 to 69 years	3.44	0.83	0.96
70 to 79 years	4.26	0.88	1.90
80 to 89 years	4.94	0.80	3.09
90 years and older	5.12	0.56	3.93
Total (30 yrs +)	3.12	0.75	0.86
t-statistics of difference between rates in a given region and those in Canada			
Atlantic Provinces	-10.39	3.48	-3.50
Quebec	21.85	-1.98	-2.80
Ontario	-5.59	-3.77	4.40
Prairies	-1.51	6.14	-1.20
British Columbia	-8.05	2.43	4.25

Table 2.8: Health care use by age according to the NPHS (1994-2011)

2.2.5 Other socio-economic variables

We constructed socio-economic variables that are useful for analysis purposes: immigrant status and gender indicators, as well as three dummy variables that combine to indicate the highest education level attained. For more information on these, see section 2.2.5 of the full technical documentation.

2.3 Canadian Community Health Survey: Description and construction of the variables

We use the Public Use Microdata Files (PUMFs) of the CCHS (Statistics Canada, 2008, 2010). Two waves of this survey are used to create the initial population in 2010, aged 30 to 110 years. We cannot use the NPHS to this end because it is only representative of the Canadian population in 1994.

The 2008-2009 and 2010 CCHS share many variables with the NPHS, which we are able to use directly. We thus have access to all the variables regarding the presence of the diseases considered in the model, with the exception of Alzheimer's and other dementias. We expand in the next chapter on the problems caused by the absence of those questions. Table 2.9 presents disease prevalence in the CCHS (2008-2009 and 2010 combined). With the exceptions of hypertension and cancer, the questions asked are limited to the presence of the health condition at the time of the interview. The question used for hypertension is retrospective, which means that we can build lifetime prevalence for this health condition. The difference between prevalences in the NPHS and in the CCHS can therefore be explained by two elements. First, as stated in the previous section, with the NPHS we recode the variables to treat three health conditions as absorbing states, something we cannot do with the CCHS for diabetes and lung disease. This explains the higher prevalence found in NPHS data for those two health conditions. Second, we use lifetime prevalence variables in the CCHS for hypertension, which explains its higher measured level when using CCHS rather than NPHS data. Prevalence for cancer, heart disease and stroke are similar in both surveys.

As observed in the NPHS, the prevalence of most diseases increases with age. The prevalence of diabetes increases up to 79 years and then decreases slightly afterwards. The most common disease is hypertension, with a prevalence of 44.2% among persons aged 60 to 69 years.

More information on other variables used from CCHS 2008-2009 and 2010 can be found in section 2.3 of the full technical documentation.

Age	Diabetes	Hypertension	Cancer	Heart disease	Stroke	Lung disease
30 to 39 years	1.9%	4.3%	0.3%	0.7%	0.15%	0.8%
40 to 49 years	3.9%	11.9%	0.9%	1.9%	0.47%	2.3%
50 to 59 years	8.6%	23.7%	1.9%	5.7%	0.78%	4.3%
60 to 69 years	14.2%	38.4%	3.8%	11.8%	2.19%	6.3%
70 to 79 years	18.7%	50.9%	6.5%	20.6%	3.61%	8.4%
80 years and older	15.4%	53.2%	6.5%	29.6%	5.36%	9.2%
Total (30 yrs +)	8.5%	28.4%	8.3%	7.5%	1.5%	4.1%
t-statistics of difference between rates in a given region and those in Canada						
Atlantic Provinces	-6.23	-9.73	-4.15	-5.98	-2.72	-4.45
Quebec	2.10	3.84	2.70	-2.66	1.88	-1.11
Ontario	-2.70	-1.93	1.09	0.05	0.46	1.91
Prairies	1.77	0.41	-0.43	2.30	-2.12	0.66
British Columbia	4.66	4.48	-2.61	5.38	0.69	0.27

Table 2.9: Self-reported disease prevalence in the CCHS (2008-2009 and 2010 combined)

Chapter 3

Initial population

Initialization involves creating a database for the initial simulation year (e.g. 2010). We cannot use the NPHS as the initial database because it was designed to be representative of the Canadian population at the time of sampling (1994). Therefore, it is not representative of the population's state of health in 2010.

We thus use a combination of the CCHS 2008-2009 and 2010 as the database to construct the initial population. These surveys have the advantage of giving an up-to-date picture (in 2010) of the state of health of the Canadian population. We use two waves in order to have a greater number of observations. The disadvantage of the CCHS (2008-2009 and 2010) compared to the NPHS is that, as opposed to the latter, the former are not longitudinal in nature. This feature, however, is not required to create an initial database.

Using a combination of surveys introduces a difficulty: the initial database must contain all the variables needed for the transitions and health care use models. For most of the variables that we use, this is not a problem since the NPHS and CCHS variables are identical. However, in a few cases, we need to impute values for certain variables that are missing from the CCHS. Moreover, we rely on the public-use version of the data, which means that the level of detail is limited for certain variables.[1] This chapter describes the assumptions that allow us to create the initial database — and thus population — using the 2008-2009 and 2010 CCHS. For the remainder of this chapter, unless otherwise stated, "CCHS" refers to the combination of CCHS 2008-2009 and 2010.

3.1 Imputation of variables and calibration

This section describes imputation procedures. Imputation involves a random process, so we create 100 initial datasets as described below to account for the randomness of the processes. We then use a different initial database for each replication of the model. This procedure is linked to the global treatment of uncertainty in the model, described in section 9.1.

[1] We rely on public-use CCHS data to construct the initial database to ensure that we can initialize the model at will outside the walls of a Statistics Canada Research Data Centre (RDC). Using restricted-access data would mean that the user would be forced to run the model within a RDC, which is not the case with the current setup.

3.1.1 Imputations

A number of variables need to be imputed; we summarize the process here. More detailed information on the foregoing can be found in section 3.1 of the full technical documentation.

- In public-use CCHS data, age is only available by 5-year group until 79 years old, and with an open category thereafter. We impute a precise age in each age group using the 2011 Census.
- Three education levels are available in the public-use CCHS.In order to have similar levels to those of the NPHS, we impute university degrees among individuals who have "post-secondary education" in the CCHS.
- The CCHS does not include individuals living in institutions. We therefore impute the institutionalization status.
- Help received for home care is present in the public-use data of CCHS 2008-2009, but not in CCHS 2010. The information is nonetheless available in the restricted-access files for CCHS 2010, so we impute it using these files.
- Alzheimer's and other dementias are the only health condition in COMPAS that cannot be observed in the public-use CCHS. We therefore impute the variable using a logistic regression estimated with 2010-2011 NPHS data.

3.1.2 Calibration of the population

A final adjustment is required to have an initial population that is representative of the 2010 population. Indeed, the size of the institutionalized population obtained through imputation differs from that provided in the 2011 Census (Statistics Canada, 2011).

In order to align the number of individuals in institutions in the initial population with the number of institutionalized individuals counted in the 2011 Census[2], we calibrate the CCHS weight with aggregated Census data. The weights are adjusted so that the total population by region, gender and age is consistent with the Canadian population aged 30 and over in 2010 (Statistics Canada, nda).

More information on calibration can be found in section 3.1.7 of the full technical documentation.

3.2 Characteristics of the initial population

After the imputations performed on 2008-2009 and 2010 CCHS data, the initial population of the model contains all the variables needed for the transitions and health care use models. Table 3.1 presents certain statistics regarding the variables of COMPAS.

As the table shows, the average age in the initial population is about 53 years (as a reminder, individuals under 30 years are excluded) and the proportion of men and women is similar. After the imputation, we find that 25% of individuals hold a university degree, which is similar to the proportion

[2]The category of accommodation we use is that of health facilities and related institutions, from which we withdraw "retirement homes".

	Average	Minimum	Maximum
Age	53.2	30	100
Immigrant status	25.4%	0	1
Women	51.3%	0	1
Less than high school	18.72%	0	1
High school graduate	22.10%	0	1
College degree	33.43%	0	1
University degree	22.75%	0	1
Presence of diabetes	8.6%	0	1
Presence of hypertension	28.9%	0	1
Presence of heart disease	7.7%	0	1
Presence of stroke	1.4%	0	1
Presence of cancer	2.3%	0	1
Presence of lung disease	4.2%	0	1
Presence of dementia	1.2%	0	1
Never smoked	35.6%	0	1
Current smoker	21.1%	0	1
Former smoker	43.37%	0	1
No obesity	79.6%	0	1
Class I obesity	14.6%	0	1
Classes II and III obesity	5.7%	0	1
Disability: no disability	90.18%	0	1
Disability: presence of cognitive impairment	1.6%	0	1
Disability: presence of at least one IADL limitation	8.5%	0	1
Disability: presence of at least one ADL limitation	2.9%	0	1
Long-term care: none	90.8%	0	1
Long-term care: home care	7.8%	0	1
Long-term care: living in an institution	1.4%	0	1

Table 3.1: Description of the initial population (mean of 100 replications)

of individuals with a university degree in the NPHS. The prevalence of diseases is below 10%, excluding hypertension which affects more than 28% of individuals. Only 1.4% of the initial population suffers from dementia.

As for risk factors, a little more than 20% of the initial population smokes and 20% of individuals suffer from obesity. The proportion of individuals who have a cognitive impairment, at least one ADL limitation or at least an IADL limitation is 9.6%. The proportion of individuals receiving home care is 7.8% while about 1% of individuals are institutionalized.

Chapter 4

Health status transitions

Using the transitions models, we can calculate the probability that the health status of individuals, in terms of diseases and risk factors, changes as a function of their individual characteristics. Such models require estimates of the parameters of discrete choice econometric models, with the transitions that we want to model serving as dependent variables.

In COMPAS, these parameters are estimated using data from the NPHS because its longitudinal nature makes it possible to observe transitions from one state to the other. The estimated parameters are then used to calculate the transition probabilities of the simulated individuals as a function of their individual characteristics and thereafter to simulate the future evolution of their health conditions.

In section 4.1, we present the econometric models used to estimate transitions in COMPAS. They estimate the effects of individual characteristics on transitions occurring between cycles of the NPHS. By default, these are two-year cycles, so the individual probabilities of changing state of health are calculated for two-year intervals.

4.1 Econometric models

4.1.1 Models for diseases

We use a transitions model to estimate incidence probabilities for each disease under consideration. The probabilities are a function of age, of risk factors, of socio-economic characteristics and, in certain cases, of the presence of other diseases. Restrictions imposed on the latter include the fact that certain diseases do not impact the incidence of other diseases, which prevents the creation of unwarranted links between them. The restrictions were evaluated by a panel of experts in the context of the Future Elderly Model (FEM) and are based on medical research on the relationship between various diseases (Goldman et al., 2005).

Table 4.1 shows the various effects permitted in the model. For example, an “ × ” at the intersection of the row “Diabetes” and the column “Hypertension” means that we allow the presence of diabetes to have an effect on the probability of getting hypertension. Moreover, diabetes, hypertension, lung disease and dementias are considered "absorbing” states. This means that once an individual suffers

from one of these diseases, he or she has it until the end of his or her life (see explanations on prevalence provided in chapter 2). For the three others conditions (cancer, heart disease and stroke), we model remission transitions.

	Diabetes	Hypertension	Cancer	Lung disease	Heart disease	Stroke	Dementias
Diabetes	n.a.	x			x	x	
Hypertension		n.a.			x	x	
Cancer			n.a.			x	
Lung disease				n.a.			
Heart disease					n.a.	x	
Stroke						n.a.	
Dementias							n.a.

Table 4.1: Permitted effects of diseases (rows) on other diseases' incidence (columns)

The specification is as follows:

$$inc^*_{i,j,t+1} = \boldsymbol{\lambda}_j \mathbf{y}_{i,t} + \boldsymbol{\beta}_j \mathbf{x}_{i,t} + \epsilon_{i,j,t} \tag{4.1}$$

with

$$inc_{i,j,t+1} = \begin{cases} 1 & \text{if} \quad inc^*_{i,j,t+1} > 0 \\ 0 & \text{if} \quad inc^*_{i,j,t+1} \leq 0 \end{cases} \tag{4.2}$$

where:

- $inc^*_{i,j,t+1}$ is a latent variable for the incidence of disease j for the individual i in period $t+1$;
- $inc_{i,j,t+1}$ is a binary variable indicating the presence of disease j for the individual i in period $t+1$;
- $\boldsymbol{\lambda}_j$ is a vector that includes the effects of the various diseases on the probability of incidence of disease j, accounting for the permitted links described in table 4.1;
- $\mathbf{y}_{i,t}$ is a vector of binary variables indicating the presence or absence of each disease ($j = 1, ..., 7$) in the individual i in period t;
- $\mathbf{x}_{i,t}$ includes all explanatory variables accounted for;
- $\boldsymbol{\beta}_j$ includes the effects of these variables on disease j;
- $\epsilon_{i,j,t}$ is a random term specific to the individual i and disease j in period t.

We estimate the parameters only on the population that, in period t, does not yet have disease j. We assume that the distribution function of $\epsilon_{i,j,t}$ follows an extreme value distribution so we can use a complementary log-log model (Sueyoshi, 1995). This model differs from the discrete choice logit and probit models, which are more commonly used, in that it relaxes the assumption that $\epsilon_{i,j,t}$ is symmetric around 0. It is therefore a better specification for cases in which the occurrence of the dependent variable is rare. The estimated parameters are used to compute the individual transition

probabilities in the simulation using the following formula:

$$\hat{P}(inc_{i,j,t+1} = 1|\mathbf{y_i}, \mathbf{t}, \mathbf{x_i}) = 1 - \exp(-\exp(\boldsymbol{\lambda}_j \mathbf{y}_{i,t} + \boldsymbol{\beta}_j \mathbf{x}_{i,t})). \tag{4.3}$$

Table 4.2 presents the average marginal effects for each disease. The first two effects are age effects, constructed as following example:

- up to 60 years: $min(age, 60)$;
- over 60 years: $max([age - 60], 0)$.

The above example applies to diabetes, hypertension, cancer and heart disease, for which a spline at 60 years old is imposed — meaning that the effect of age in the model is allowed to change after 60 years. We use splines at different ages for the different conditions, either at 45, 60 or 85 years old. The ages were chosen for each disease by testing which one had the highest explanatory power.

Smoking positively and significantly affects the probability of incidence of certain diseases, such as hypertension, heart disease, stroke and lung disease. Being a former smoker also positively impacts the probabilities of incidence, but less so than currently smoking. Smoking does not significantly affect the likelihood of developing cancer, which may seem counter-intuitive as 13.5% of all diagnosed cancers are lung cancers (Canadian Cancer Society's Advisory Committee on Cancer Statistics, 2015). One possible explanation is the very short life expectancy of lung cancer patients: only 32% survive one year after their diagnosis (Cancer Research UK, nd). Individuals are surveyed only at two-year intervals, so many might be diagnosed and die between two survey cycles.

Obesity has a generally positive effect on the probabilities of disease incidence, with the exception of Alzheimer's and other dementias, and of stroke (for classes II and III obesity).

Women seem less affected by diabetes and heart disease and more affected by lung disease. As for immigrants, they have less of a chance of being affected by heart and lung disease.

A higher educational attainment is generally associated with lower incidence, with the exception of cancer. The estimated effects of education may capture two effects that might counter one another. Better educated individuals are, on the one hand, generally healthier but, on the other hand, more likely to consult with a health professional, which might increase their disease incidence.

The dummy variables for province of residence are not significant in most cases, with the exception of Prairie residents for the incidence of diabetes and dementia. However, they are statistically significant when tested all together (F-test).

The effects of the presence of other diseases on disease incidence are generally positive and significant when we allow for non-null effects. The exceptions are that the presence of cancer or hypertension does not seem to significantly affect the probability of stroke incidence.

The same variables as in incidence models are used in remission models. Age has a negative effect on the probability of remission before 60 years old, and a positive effect after that for both heart disease and cancer. Smoking significantly increases the probability of remission from cancer, which may seem counter-intuitive; but it must be reminded that this is the remission rate among survivors for at least 2 years following diagnosis, and that many patients likely die without having been re-surveyed. Women have better chances of remission, as do people with a college or university degree. Residing in any

	Diabetes	Hypertension	Cancer	Heart disease
Age (if 60 years or under)	0.0009***	0.0032***	0.0011***	0.0021***
Age (if over 60 years)	0.0001	0.0000	0.0003**	0.0014***
Current smoker	0.0012	0.0081**	0.0025	0.0111***
Former smoker	0.0013	0.0044	0.0012	0.0086***
Class I obesity	0.0144***	0.0250***	−0.0004	0.0038
Classes II-III obesity	0.0258***	0.0405***	0.0048*	0.0116***
Woman	−0.0038**	−0.0020	0.0009	−0.0076**
Immigrant	0.0004	0.0063	−0.0021	−0.0078***
High school graduate	0.0007	−0.0058*	0.0019	−0.0018
College degree	−0.0001	−0.0024	0.0033	−0.0027
University degree	−0.0058**	−0.0099*	−0.0001	−0.0034
Resides in Quebec	−0.0012	−0.0013	−0.0032	0.0022
Resides in Ontario	−0.0008	−0.0014	0.0011	0.0059**
Resides in the Prairies	−0.0048**	−0.0051	−0.0003	−0.0015
Resides in British Columbia	−0.0020	−0.0071	−0.0017	−0.0008
Presence of diabetes	—	0.0248***	—	0.0113***
Presence of hypertension	—	—	—	0.0183***
Presence of heart disease	—	—	—	—
Presence of cancer	—	—	—	—
Number of observations	65,000	53,700	67,900	64,600
Average incidence	0.0105	0.0332	0.0087	0.0160

	Stroke	Lung disease	Dementias
Age (if 85 years or under)	0.0005***	—	0.0017***
Age (if over 85 years)	0.0005	—	0.0005
Age (if 45 years or under)	—	−0.0002	—
Age (if over 45 years)	—	0.0004**	—
Current smoker	0.0047**	0.0109***	0.0032
Former smoker	0.0007	0.0049***	0.0022
Class I obesity	−0.0013	0.0014	−0.0022
Classes II-III obesity	−0.0078*	0.0067**	−0.0064
Woman	−0.0009	0.0033**	−0.0025
Immigrant	0.0021	−0.0037*	0.0034
High school graduate	−0.0013	−0.0030*	−0.0033
College degree	−0.0023	−0.0029	−0.0044
University degree	−0.0086***	−0.0048*	−0.0086**
Resides in Quebec	−0.0013	0.0012	0.0044
Resides in Ontario	0.0006	0.0006	0.0041
Resides in the Prairies	0.0010	−0.0004	0.0074**
Resides in British Columbia	−0.0006	−0.0015	0.0024
Presence of diabetes	0.0062***	—	—
Presence of hypertension	0.0024*	—	—
Presence of heart disease	0.0055***	—	—
Presence of cancer	−0.0001	—	—
Number of observations	52,100	64,600	28,800
Average incidence	0.0054	0.0088	0.0053
Legend	* p <0.10 ; ** p <0.05 ; *** p <0.001		

Table 4.2: Average marginal effects of variables on the probabilities of disease incidence over two years

Variable	Cancer	Heart Disease	Stroke
Age (if 60 years or under)	−0.0036	−0.0053***	—
Age (if over 60 years)	0.0014	0.0029**	—
Age (if 85 years or under)	—	—	0.0002
Age (if over 85 years)	—	—	0.0032
Current smoker	0.1272*	0.0152	−0.0584
Former smoker	0.0445	−0.0152	0.0080
Class I obesity	0.0763	−0.0287	0.0568
Classes II-III obesity	−0.0710	0.0122	−0.0189
Woman	0.0844*	0.0235	0.1227**
Immigrant	−0.0433	0.0264	−0.0046
High school graduate	−0.0003	0.0036	−0.0069
College degree	0.0739	0.0266	0.0099
University degree	0.1421*	0.0195	−0.1947*
Resides in Quebec	0.1575**	0.0607*	−0.0542
Resides in Ontario	0.0941	0.0170	−0.0649
Resides in the Prairies	0.0583	0.0419	−0.0649
Resides in British Columbia	0.1669**	0.0084	−0.0308
Presence of diabetes	—	0.0005	0.0034
Presence of hypertension	—	−0.0519**	0.0286
Presence of heart disease	—	—	−0.0231
Presence of cancer	—	—	0.0025
Number of observations	65,000	53,700	67,900
Legend	* p <0.10 ; ** p <0.05 ; *** p <0.001		

Table 4.3: Average marginal effects of variables on the probabilities of disease remission

region West of the Maritime provinces generally increases the probability of remission, or leaves it unchanged. The presence of hypertension reduces the probability of remission from heart disease.

4.1.2 Model for mortality

The mortality model is similar to that for disease incidence, but we include additional explanatory variables: disability statuses and whether the person is living in a long-term care facility. Moreover, we do not impose any restrictions on the effects of the various diseases' presence.

Table 4.4 presents the marginal effects of the variables on the probability of dying in the two subsequent years. Unsurprisingly, the probability of dying increases significantly with age, both before and after 50 years of age. It may seem surprising, however, that the increase slows down after 50 years, but this means that death at older ages is better predicted by other variables in the model that are themselves correlated with age. Indeed, the presence of diseases positively affects the probability of death, and this relationship is statistically significant in many cases (diabetes, cancer, heart disease).

Being a current or a former smoker also significantly affects the probability of death. The link between obesity and death is negative: this can be explained by the weight loss that often occurs in the last stages of life.

Age (if 50 years or under)	0.0018***
Age (if over 50 years)	0.0010***
Presence of diabetes	0.0054**
Presence of hypertension	0.0011
Presence of cancer	0.0186***
Presence of heart disease	0.0054***
Presence of stroke	0.0032
Presence of lung disease	0.0013
Presence of dementia	−0.0008
Current smoker	0.0145***
Former smoker	0.0068***
Class I obesity	−0.0044*
Classes II-III obesity	−0.0031
Woman	−0.0083***
Immigrant	−0.0011
High school graduate	−0.0041**
College degree	−0.0056**
University degree	−0.0070***
Resides in Quebec	0.0002
Resides in Ontario	0.0009
Resides in the Prairies	−0.0020
Resides in British Columbia	0.0013
Presence of cognitive impairment only	0.0133
Presence of IADL limitations only	0.0153***
Presence of ADL limitations only	0.0093
Presence of cognitive impairment and IADL limitations	0.0192*
Presence of ADL and IADL limitations	0.0284***
Presence of cognitive impairment and of ADL and IADL limitations	0.0476***
Living in a long-term care facility	0.0052
Number of observations	83,261
Average mortality	1.98%
Legend : * p <0.10 ; ** p<0.05 ; *** p <0.001	

Table 4.4: Average marginal effects on two-year probability of death

Being a woman decreases the probability of death, which is consistent with the higher life expectancy of this gender. Higher educational attainment also leads to a significant decrease in the probability of death.

As discussed in chapter 2, mortality rates observed in the NPHS are slightly different from official mortality rates, especially at higher ages. As a result, the number of deaths per year in our model could be biased. We calibrate the estimated probabilities to fit the mortality rates in the Human Mortality Database, which reflect the mortality rates provided by Statistics Canada.

We calibrate mortality at the first year of simulation. To do so, we first estimate the average probability of death by age and gender from the 100 starting populations. We subsequently compute an adjustment factor by age and sex, equal to the rate estimated using the moving average divided by the mortality rates derived from the Human Mortality Database. Finally, the relevant adjustment factor is applied to the calculated probability of death in each simulation year.

4.1.3 Models for smoking

We estimate three transition models for smoking:

1. Smoking initiation (estimated for individuals who have never smoked);
2. Smoking cessation (estimated for current smokers);
3. return to smoking (estimated for former smokers).

Once again, we use a complementary log-log model for all three transitions. Even if the initial population is 30 years and older we model smoking initiation, as we observe some such transitions in the NPHS data. The presence of disease is, however, excluded from the explanatory variables since smoking is considered a risk factor. Indeed, smoking increases the probability that individuals develop certain diseases and then die as a consequence. For more details on the models for smoking, see section 4.1.3 of the full technical documentation.

4.1.4 Model for obesity

We model the transitions between the various possible states of obesity (BMI < 30, $30 \leq$ BMI < 35, and BMI ≥ 35) using a multinomial logit model. The model does not include the presence of disease and states of disability as explanatory factors, since obesity is considered a risk factor that affects these variables as opposed to a consequence of them. For further details on the models for obesity, see section 4.1.4 in the full technical documentation.

4.1.5 Model for disability

We model the transition probabilities between all the states of disability allowed for in the model. The three states in which we are interested are the presence of cognitive impairment, the presence of at least one limitation in activities of daily living (ADL) and the presence of at least one limitation in instrumental activities of daily living (IADL). We then create all possible combinations of these three states. Therefore, each individual can be in one of eight combinations of states ($2 \times 2 \times 2$, since each state involves two possible values — presence or absence). For more details on the disability model, see section 4.1.5 of the full technical documentation.

4.1.6 Model for long-term care

We model the probability of transitioning between different states of long-term care (LTC) use. There are three possible states: no LTC use, receiving home care, or residing in a LTC facility. Institutionalization thus characterizes both disability and use of LTC in the model. As with the model for disability, we use a multinomial logit model, which estimates the probability for an individual to end up in each of the possible states of LTC use in the next cycle. For more details on the long-term care model, see section 4.1.6 of the full technical documentation.

4.2 Transitions validation

To validate the quality of our estimates, we simulated the evolution of the population from 1994 to 2010 using the previously estimated parameters and compared the characteristics of that population with those obtained in the actual NPHS population: we started with the population of the 1994 wave, and simulated the first transitions. We used the simulated population of 1996 and simulated the transitions, and so on, until 2010. With incorrect dynamics, we would end up with very different disease prevalences in 2010. The results are shown in Table 4.5. Although there are differences, the results are generally satisfactory.

	Diabetes	Hypertension	Cancer	Heart disease	Stroke	Lung disease	Dementias
1994	0.0000	0.0000	0.0000	0.0000	0.0000	0.0000	0.0000
1996	0.0016	-0.0052	0.0003	-0.0083	-0.0001	-0.0055	0.0014
1998	0.0064	-0.0014	0.0016	-0.0079	0.0014	-0.0080	0.0042
2000	0.0073	-0.0031	0.0017	-0.0085	0.0019	-0.0104	0.0067
2002	0.0076	-0.0079	0.0057	-0.0058	0.0018	-0.0132	0.0099
2004	0.0082	-0.0025	0.0106	-0.0025	0.0047	-0.0114	0.0118
2006	0.0100	0.0049	0.0148	0.0075	0.0109	-0.0076	0.0161
2008	0.0023	-0.0087	0.0105	0.0046	0.0125	-0.0129	0.0163
2010	0.0073	0.0046	0.0148	0.0159	0.0162	-0.0099	0.0184
1994-2010	0.0062	-0.0015	0.0069	-0.0003	0.0053	-0.0080	0.0092

Table 4.5: Difference in prevalences between simulated population and NPHS population — 1994-2010

A second validation is done by comparing the projections of period life expectancy obtained in COMPAS with the ones produced by Statistics Canada (Bohnert et al., 2015).[1] The results from COMPAS are close to official projections. In the 2050, the 90% confidence interval for COMPAS projections lies between Statistics Canada's Low and High projection scenarios, respectively.

	Life expectancy at 30 years old			
	StatsCan-Low	StatsCan-M1	StatsCan-High	COMPAS
2030	54.1	54.9	56.2	54.8 (53.6, 56.0)
2050	55.9	57.3	59.4	57.5 (56.5, 58.7)
	Life expectancy at 65 years old			
	StatsCan-Low	StatsCan-M1	StatsCan-High	COMPAS
2030	21.5	22.0	23.0	21.7 (20.6, 22.8)
2050	22.7	23.7	25.4	23.9 (23.0, 25.0)

Table 4.6: Comparison of projected life expectancies for the province of Quebec
Source: COMPAS, Statistics Canada (ndb) and authors' calculations.
Note: 5th and 95th percentiles of estimates shown in brackets.

[1]The projections by province published by Statistics Canada stop in 2038, so we extend the projection for Quebec until 2050. Because they are not publicly available, we have obtained the detailed projections of mortality by age, sex, province and year used in preparing Population Projections for Canada (2013-2063) directly from Statistics Canada to reconstruct the period life expectancies.

Chapter 5

Population renewal

In COMPAS, entry of a new cohort of individuals aged 30 and 31 years is implemented in each simulation cycle, that is every 2 years by default. Each entering individual has many characteristics. In proportion, these should at all times reflect the joint distribution of the initial conditions (at $t = 0$) of the target population aged 30 and 31 years, as well as potential trend changes that we will have applied. In the full technical documentation the reader can find a detailed exposition of the modelling framework (section 5.1), a description of the historical trends used to construct the projection trends (section 5.2.1), and the explanation of the characteristics of new cohort from 2010 to 2050 (section 5.2.2).

Chapter 6

Demography: Mortality and immigration

The present chapter describes the assumptions with regard to mortality and immigration, as well as the demographic models which serve as the basis for COMPAS. It also describes the modelling of mortality improvements.

6.1 Mortality

6.1.1 Definitions and statistics

Historical data shows a clear improvement in mortality rates between 1960 and 2011. Life expectancy rose from 68.2 to 79.5 years for men and from 74.2 to 83.7 years for women during this period (Human Mortality Database, 2005). In COMPAS, given that young cohorts aged 30 and 31 years enter the modelling process up until 2050, the very efficiency of our model requires forecasts of mortality rates over a long period. Moreover, given that an individual may reach 110 years old in COMPAS, the forecasts should ideally include age groups for the oldest among the elderly, or those aged 90 and over.

Improved overall mortality depends mainly on two factors: *(i)* trends linked to diseases and risk factors; and *(ii)* technological progress, particularly in medicine. The contribution of diseases and risk factors is largely modelled in COMPAS even though, as stated in chapter 4, the transition models are not a function of time. The overall combined effect of diseases and risk factors is nearly nil in COMPAS as a result of the various opposing effects. Thus, in the long term, the significant improvement in mortality mostly comes from technological progress. As technological progress is not modelled in COMPAS, we rely on exogenous assumptions with respect to mortality rate improvement.

Hereafter we present the sources of our mortality rate forecasts.

6.1.2 Estimation methods and integration into COMPAS

We use the latest Statistics Canada projections of mortality rates from Population Projections for Canada (2013-2063) (Statistics Canada, 2015). These projections include all the characteristics needed for our simulations. They go to 2063 and they are finely defined by age, sex and province (they are available by province up to 2038).[1] Projections are provided for old ages, up to 110 years old.[2]

We use the mortality table "as is", with three exceptions. First, we group provinces in five regions (Atlantic, Quebec, Ontario, Prairies, British Columbia) by simply calculating the weighted mean of the provincial mortality rates by age, sex and year. Second, we apply to all regions the Canadian evolution of mortality for the period after 2038. Third, we only use Statistics Canada projections until 2050; after this point we keep mortality rates constant at 2050 rates. Table 6.1 shows the annual reduction of mortality rates based on Statistics Canada's forecasts. Hereafter, we present the integration of the prospective mortality table into the COMPAS model.

Age group	2013-2019	2020-2029	2030-2039	2040-2050
		Men		
30 to 39	2.1%	2.1%	2.1%	2.2%
40 to 49	2.1%	2.2%	2.2%	2.2%
50 to 59	2.6%	2.5%	2.4%	2.4%
60 to 69	2.7%	2.6%	2.5%	2.4%
70 to 79	2.3%	2.2%	2.1%	2.1%
80 to 89	1.1%	1.1%	1.0%	1.0%
90 to 99	0.3%	0.3%	0.2%	0.2%
100 to 110	0.2%	0.1%	0.1%	0.1%
		Women		
30 to 39	1.9%	1.7%	1.7%	1.7%
40 to 49	1.8%	1.7%	1.7%	1.7%
50 to 59	1.8%	1.8%	1.8%	1.8%
60 to 69	1.8%	1.8%	1.8%	1.8%
70 to 79	1.7%	1.6%	1.6%	1.6%
80 to 89	1.0%	0.9%	0.9%	0.9%
90 to 99	0.3%	0.2%	0.2%	0.2%
100 to 110	0.2%	0.1%	0.1%	0.1%

Table 6.1: Annual rates (in %) of mortality rates reduction.
Source: Statistics Canada (2015) and authors' computation.

The model used by Statistics Canada does not incorporate opinions on the potential evolution of mortality due to advances in medicine, to the appearance of new illnesses or risk factors or to changing lifestyles. In this respect, it is worth noting that Statistics Canada explicitly recognizes that its projections are based on an extrapolation of past trends: *"More than any other component of demographic growth, mortality lends itself to projections based on the extrapolation of past data"* (Bohnert et al., 2015, p.42). Now, in COMPAS we can build scenarios to model advances in medicine by changing the mortality projections, changes in risk factor exposure or modifications in the incidence of diseases.

[1] Because they are not publicly available, we have obtained the detailed projections of mortality by age, sex, province and year used in preparing Population Projections for Canada (2013-2063) directly from Statistics Canada.

[2] For more information on the methodology used by Statistics Canada, see Bohnert et al. (2015).

These "official" projections are integrated into COMPAS as exogenous mortality improvements by simply applying to the individual mortality probability at each period of simulation an adjustment factor computed by age, sex, province and year of projection. An exhaustive presentation of this process is available in section 6.1.3 of the full technical documentation.

6.2 Immigration

In order to correctly reflect demographic reality, we should account for individuals moving from one country to another — *international migration* — or from one province to another — *interprovincial migration*. A description of how migrations are taken into account can be found in section 6.2 of the full technical documentation.

6.3 Conclusion

By integrating the mortality model and the migratory balance forecasts in COMPAS and combining the results with Statistics Canada's forecasts for individuals under 30 years old,[3] we obtain a total population for Canada of just over 45 million inhabitants in 2050. As shown in Table 6.2, the population forecast by COMPAS lays between Statistics Canada's "low" and "medium" projections, with a handful of age groups just under the population of the "low" scenario in 2016 and 2030. The population projected by COMPAS shifts from Statistics Canada's "low" scenario towards its "medium" one as the simulation progresses in time.

[3]This operation is required here because COMPAS only simulates the population aged 30 and over.

	COMPAS			StatsCan-Low			StatsCan-M1		
Age group	2016	2030	2050	2016	2030	2050	2016	2030	2050
0 to 30	12.84	13.66	15.31	12.72	12.43	12.15	12.84	13.66	15.31
30 to 39	4.75	4.87	4.90	4.91	4.91	4.87	5.0	5.3	5.8
40 to 49	4.76	5.21	5.33	4.76	5.09	4.79	4.8	5.4	5.7
50 to 59	5.35	4.90	5.41	5.36	4.72	4.97	5.4	4.9	5.7
60 to 69	4.31	5.07	5.30	4.27	4.86	4.85	4.3	5.0	5.4
70 to 79	2.50	4.30	4.57	2.46	4.10	4.10	2.5	4.2	4.4
80 to 89	1.28	2.19	3.65	1.24	2.07	3.11	1.2	2.1	3.4
90+	0.32	0.45	1.24	0.29	0.42	0.98	0.3	0.4	1.1
All ages	36.1	40.6	45.7	36.0	38.6	39.8	36.2	41.1	46.9

Table 6.2: Comparison of projected population for Canada: COMPAS vs. Statistics Canada
Source: COMPAS, Statistics Canada (ndb) and authors' calculations.
Note: Figures for the 0-30 years old in COMPAS are those from the Statistics Canada M1 forecast.

Chapter 7

Health care use

The health care use module makes it possible to evaluate, for each simulation year, the quantity of medical resources used by the population. In order to obtain these results, however, we must first establish a relationship between health status and resource use. This chapter describes the process; in COMPAS, the parameters presented here are then applied to the projected population at the end of each simulation cycle.

COMPAS includes several measures of health care use. We model separately the number of consultations with a generalist physician and with a specialist physician; the number of nights an individual is hospitalized; the consumption of drugs; the use of formal and informal home care; and the number of hours used of both types of home care.

7.1 Modelling

This section presents the data and variables used in estimating health care use and briefly explains the econometric models used. We rely on the NPHS (see chapter 2 on this topic) for all the health care use estimation, with the exception of the number of hours of formal and informal home care. For those, we use the General Social Survey (GSS) of 2012 (cycle 26). With the NPHS, we only use data from 2000-2001 to 2010-2011 to control for the risk that health care use patterns may have changed over time. While the regression type (i.e. the econometric model) used differs depending on the category of health care considered, explanatory variables remain the same in all regressions that use NPHS data. These regressions are only performed using respondents who live in private households in the current period, because there is insufficient information on health care use for those living in an institution. For example, if a respondent is in an institution at the time of the last survey cycle, his/her answers to the preceding cycles are still used (only responses from the last cycle are excluded).

The socio-demographic independent variables used in NPHS-based regressions are sex, age, immigrant status, education, and indicators for respondents living in each Canadian region (Atlantic, Quebec, Ontario, Prairies, and British Columbia). As elsewhere in this report, Atlantic is the reference. The effect of age on resource use does not appear to be linear. Specifically, it appears to change after 50 years of age. In order to capture this effect, a spline was created (Goldman et al., 2005). There

are therefore two age variables, one for individuals under the age of 50 and another for those aged 50 years and over. This last variable represents the number of years since the individual turned 50 (e.g., it takes a value of 5 for a 55-year-old person). The estimated coefficient of the first age variable thus captures the effect of age up to 50 years on the dependent variable; similarly, the coefficient of the second variable is the effect of age after 50 years on the use of health care resources. We use three variables for disability (1+ cognitive disability, 1+ IADL, and 1+ ADL) and dummies for the presence of the seven diseases included in COMPAS. Tobacco use is included with two binary variables: one indicating former smokers and one for current smokers. Two binary variables are included in the regression to capture the effect of class I ($30 \leq$ BMI <35) and classes II-III (BMI ≥ 35) obesity. The reference categories for risk factors are thus individuals who have never smoked and those with a BMI under 30.

Regressions using GSS data rely on fewer variables as we need to use variables that have counterparts in the CCHS. We use sex, age and Canadian regions in the same format as in NPHS-based regressions. We include the seven diseases but heart disease, stroke and hypertension are grouped into cardiovascular diseases because only one question is asked in GSS for these three diseases combined. We do not include disability variables, because questions are relative to help received for a disability. The questions are too far from those used in the CCHS and NPHS to include disability variables in our regression.

Consultations with physicians, number of nights hospitalized and hours of home care services are generally characterized by a large number of observations with missing values (or small values used) and an asymmetric distribution. Stated otherwise, the foregoing means that during a reference period, many individuals do not actually use any of these medical resources while others use much more than the average (Frees et al., 2011). In order to account for such a distribution of data, we use a negative binomial regression.

For drug consumption, which in the current version of the model can take two values (”yes” or “no”), a logistic regression is used. Use of home care services can take three values (informal, formal or both) conditional on using any home care, so we use a multinomial logistic model, the framework for which is presented in section 4.1.5 of the full technical documentation. We present the logistic regression and the negative binomial regression hereafter. The econometric theory of the models is based on Cameron and Trivedi (2005).

7.1.1 Negative binomial regression

The negative binomial regression is generally used to analyze discrete and countable data, i.e. data that only take whole and non-negative values, which fits well with health care use data. This type of regression, by allowing for over-dispersion of data (variance higher than the mean), can capture the asymmetry in the distribution observed in lots of health care use data. Using the Poisson distribution, which assumes equality between mean and variance, would not have matched the distribution of the data as effectively.

The negative binomial regression assumes that the observations are generated from a negative binomial distribution, the first two moments of which are:

$$\mathbb{E}(y_i|\mu_i, \alpha) = \mu_i = \exp(\boldsymbol{x}_i'\boldsymbol{\beta}) \quad (7.1)$$

$$\mathbb{V}ar(y_i|\mu_i, \alpha) = \mu_i(1 + \alpha\mu_i) \quad (7.2)$$

where:

- $\mathbf{x}_i$ is a vector of explanatory variables for individual i;
- $\boldsymbol{\beta}$ is a vector of coefficients;
- α is the over-dispersion parameter.

If $\alpha > 0$, it necessarily follows from the definition of variance that there is over-dispersion, given that the variance is greater than the mean (μ_i being positive). This definition of variance is the one used by the *nbreg* command in Stata to perform the negative binomial regression. Estimation of coefficients is done using the maximum likelihood method.

However, the estimated parameters do not directly yield the effects of a variable x on the conditional expected value of y. Thus, it is not the parameters themselves which are interesting when analyzing the explanatory variables' impact, but rather the marginal effects, namely, the change in the conditional expected value of y_i when the value of a variable x_i changes by one unit. As elsewhere in this report, we present the average marginal effect (AME), which is the average change, over all individuals, in the expected value of y_i when x_i changes by one unit.

7.1.2 Logistic regression

The logistic regression, as mentioned above, is used with variables that can only take two values, such as consumption (or not) of at least one drug. A more detailed explanation on logistic regression can be found in the full technical documentation.

7.2 Results

7.2.1 Physician consultations and hospitalizations

The effect of the different illnesses on use of medical resources is presented in table 7.1. Use is computed over a 12-month period. As such, if an individual is said to have consulted with a specialist 3 times, this indicates that he consulted with a specialist 3 times over the course of a 12-month period. As expected, illnesses have a large effect on the number of consultations with a generalist. The presence of diabetes, hypertension, cancer, heart disease or lung disease increases the number of consultations with a generalist by 0.7 to 1.2 visits. The presence of dementia has no significant effect, but the presence of cognitive impairment does. Classes I and II-III obesity also increase the number of consultations, by 0.3 and 0.6 respectively. However, it is disabilities that lead to the largest increase in consultations. An individual with at least one IADL had 1.5 more consultations on average than an individual without any IADL. On average, Quebecers consult with physicians less often than individuals in the rest of Canada; the gap is between 0.85 and 1.3 consultations over 12 months. Women also consult a generalist more often.

Variable	Nb. of consultations: generalist	Nb. of consultations: specialist	Nb. of nights hospitalized
Age (if under 50)	−0.001	0.001	−0.008
Age (if 50 years or over)	0.007*	−0.007***	0.023**
Woman	0.823***	0.244***	0.223
Immigrant	0.210*	−0.046	−0.389*
High school graduate	−0.051	0.211***	−0.144
College degree	−0.089	0.188***	−0.105
University degree	−0.069	0.366***	−0.182
Resides in Quebec	−1.106***	0.164***	0.186
Resides in Ontario	−0.246**	0.151***	−0.511***
Resides in the Prairies	−0.229**	−0.022	−0.100
Resides in British Columbia	0.193	0.086	−0.100
Presence of diabetes	0.887***	0.085*	0.478**
Presence of hypertension	0.993***	0.199***	0.434***
Presence of cancer	1.168***	1.003***	1.593***
Presence of heart disease	1.053***	0.470***	1.251***
Presence of stroke	0.693***	−0.098	1.356**
Presence of lung disease	0.665***	0.107**	−0.001
Presence of dementia	0.021	0.122	−0.175
Class I obesity	0.327***	0.051	−0.016
Classes II-III obesity	0.626***	0.148**	0.333
Current smoker	0.218*	−0.022	0.262
Former smoker	0.224***	0.078*	0.134
Presence of ADL	0.281	0.124	0.854***
Presence of IADL	1.523***	0.524***	1.726***
Presence of cognitive impairment	0.922***	0.265***	0.962**
Number of observations	49,079	49,147	43,050
Average number of consultations or nights	3.12	0.75	0.86
Legend	* p<0.05 ; **p<0.01; *** p<0.001		

Table 7.1: Average marginal effect of different variables on health care use.
Note: Use is computed on an annual basis. Binomial negative regression used.

The effect of illnesses on the number of consultations with a specialist is also positive and significant for some illnesses. The presence of hypertension, cancer, heart disease or lung disease increases the number of consultations. Classes II-III obesity increases the number of specialist consultations by 0.1 per year on average, while tobacco use variables have little effect.

The presence of dementia or lung disease does not have a significant impact on the number of nights spent in short-term hospitalization, but all other types of illnesses have considerable effects. The presence of a cancer or of stroke increases the number of nights hospitalized by just over 1 on average. Individuals suffering from disabilities spend an average of 1.2 more nights in hospital: 0.9 nights for those with ADL, 1.7 for those with IADL, and 1.0 for those with cognitive impairment. Individuals suffering from two or more disabilities spend an average of 4.2 more nights in hospital than those with no disabilities. The coefficient of class I obesity is not significant and is near zero, but individuals with a BMI of 35 or over (classes II-III) spend an average of 0.3 more nights in hospital than those who are not overweight.

7.2.2 Medications

See the full technical documentation, section 7.2.2 for more information on prescription and over-the-counter drug use modelling, which is not used in this article.

7.2.3 Home care services

Chapter 4 presented the estimation method to determine whether an individual receives home care services. We also estimate the types of services used – formal, informal or both – among individuals who receive home care, after determining whether an individual receives home care or not (see section 4.1.5). We also determine the number of hours received for each type of home care received. See section 7.2.3 of the full technical documentation for more information on home care services.

7.3 Conclusion

The main take-away from the modelling and results presented in this chapter is that the illnesses considered in COMPAS are important factors in explaining the use of health care resources. Disability also has sizeable effects. Finally, there are significant differences between Canadian regions.

Chapter 8

Health care costs

This chapter discusses the methodology used to include the costs of health care in COMPAS. At this stage of the model's development, the only costs that we can model are those for Quebec. Thus, we model the costs of hospitalization and consultations with general practitioners and specialists. To do this, we use 2012 data from the Régie de l'assurance maladie du Québec (RAMQ) and from the *Maintenance et exploitation des données pour l'étude de la clientèle hospitalière* (MED-ECHO) database, maintained by the Ministère de la Santé et des Services sociaux (MSSS).

For each health care use variable, we try to estimate the cost of the resource based on a number of characteristics such as age, gender and the presence of diseases (when the data allows). We use these estimates to calculate the cost of the health care received by each simulated individual according to his or her characteristics. We begin by describing in detail the data used to attribute costs to the health care used. We then present the models to calculate these costs.

8.1 Hospital stays: MED-ECHO data and NIRRU

To estimate the cost of hospitalizations, we match the MED-ECHO data to the RAMQ data. MED-ECHO is an administrative database that includes all available information on hospitalizations. However, it excludes stays in psychiatric hospitals, rehabilitation hospitals and long-term care facilities as well as physician salary costs. To capture physician salary costs, we must also use the RAMQ data. These are described in section 8.2. It should be noted that the MED-ECHO data does not include hospital costs related to emergency services. Furthermore, the MED-ECHO data that we have for the time being does not include the cost of hospital day surgeries.

MED-ECHO data does not directly provide the cost of hospitalization. The MSSS uses a relative intensity of resource use (*niveau d'intensité relative des ressources utilisées* or NIRRU in Quebec) paired with the MED-ECHO database. The NIRRU is an index to calculate the total cost of hospitalization, excluding physician salary costs, by measuring the amount of resources used during the hospital stay. It includes treatment and intervention costs; medicine costs; the cost of transportation, of hospital beds, of depreciation, etc. The database provided by the MSSS includes a NIRRU per hospitalization

in addition to a "cost per unit" of NIRRU (or the cost associated with a NIRRU of 1). Combining both data points gives the cost of hospitalization, except for the services provided by physicians.

The full technical documentation provides more information on MED-ECHO (section 8.1) and the NIRRU (section 8.1.1), as well as descriptive information on the length and cost of hospital stays (section 8.1.2).

8.2 Physicians: RAMQ data

Thanks to data about physician services billed to the RAMQ, we can capture the cost of the procedures performed by physicians working on fee-for-service schedules or mixed remuneration arrangements.[1] According to Boulenger and Castonguay (2012), this data covers the vast majority of Quebec physicians, with 84% of their total compensation in 2009 taking one of these forms (73% being fee-for-service and 11% mixed).

In addition to the fee charged and the procedure code of the service performed, RAMQ data includes the date of the service, the identity of the patient, and whether the service was performed by a general practitioner or a specialist. More detailed information on the RAMQ data as well as descriptive data can be found in the full technical documentation, in section 8.2.

8.3 Models

We use the models presented in this section to calculate the annual cost of hospitalizations, physician consultations, home care and institutionalization. These models are estimated using data from the RAMQ, MED-ECHO, insurance companies, CIHI and Statistics Canada. The estimated parameters are used to calculate the individual annual cost of health care use.

8.3.1 Hospitalizations

To calculate the cost of hospitalization, we estimate two models: one for men and one for women. Both models are linear, but include a polynomial of degree four for the annual length of hospital stays (LS). The LS for an individual is the sum of all nights (or days) spent in hospital during the year. The specification is as follows:

$$cost\_hospitalization_i = \lambda_1 LS_i + \lambda_2 LS_i^2 + \lambda_3 LS_i^3 + \lambda_4 LS_i^4 + \beta_i \mathbf{x}_i + \epsilon_i, \tag{8.1}$$

where:

- $cost\_hospitalization_i$ is the annual cost of hospital stays for the individual i;
- LS_i is the length of stay (in days) for the individual i;

[1] A doctor is paid on a fee-for-service basis if he or she bills the RAMQ for each service rendered. A doctor receives a mixed remuneration if he or she receives a fixed amount per day as well as a supplement for each service rendered.

- LS_i^2 is the length of stay (in days) for the individual i raised to the power of 2;
- LS_i^3 is the length of stay (in days) for the individual i raised to the power of 3;
- LS_i^4 is the length of stay (in days) for the individual i raised to the power of 4;
- $\mathbf{x}_i$ includes all other explanatory variables;
- ϵ_i is a random term specific to individual i.

The explanatory variables are limited because MED-ECHO is an administrative database. We therefore include the presence (or absence) of all diseases (excluding dementias, which are very difficult to capture in the data). We also add a dummy variable for ages 70 years and over to capture a possible effect of age.

Table 8.1 presents the estimation for the annual cost of hospitalizations. The model is estimated only among individuals who had at least one hospital stay in 2012.

	Men	Women
70 years and more	−2,391.16216***	−1,807.18420***
Stroke	−1,172.86396***	−609.38623*
Diabetes	7.78941	271.91647
Hypertension	178.74447	−173.52830
Lung disease	1,939.62067**	221.98001
Heart disease	1,959.85891***	1,736.42148***
Cancer	816.82019***	1,900.19386***
Length of stay	1,116.83700***	906.97822***
(Length of stay)^2	−11.54489***	−8.10794***
(Length of stay)^3	0.04656***	0.02849***
(Length of stay)^4	−0.00006***	−0.00003***
Number of observations	12,886	17,541
Average cost ($)	13,256	10,609
Legend	* p <0.10 ; ** p <0.05 ; *** p <0.001	

Table 8.1: Average marginal effect of the different variables on the annual cost of hospitalization

The dummy variable for age has a negative effect on the annual cost of hospitalization for both men and women. This suggests that the marginal effect captures a residual effect of age on the cost of hospitalization. Indeed, hospital stay variables and the presence of diseases are likely to capture much of this. One possible explanation is that older people might tend to have longer hospital stays because they do not have a suitable place to go to at their discharge from hospital. These additional nights cost less on average than the nights of younger individuals who owe the entire length of their stay to their health condition.

The presence of a majority of the diseases has a positive effect on the annual cost of hospitalization, except for the presence of a past stroke (men and women) and hypertension (for women only), which are associated with a lower cost. The presence of heart disease has a very important effect on the cost: for men (women), it increases the annual cost of the hospitalization by $1,959 ($1,736). Unsurprisingly, the length of stay is also positively related to the cost of hospitalization.

8.3.2 Consultations

We calculate the cost of consultations using four linear models that include a polynomial of degree four for the number of consultations (NC) in the last 12 months. The first estimates the cost of visits to a general practitioner and the second estimates the cost of consultations with a specialist. The model specification is as follows:

$$cost\_consultation_{i,j} = \lambda_1 NC_i + \lambda_2 NC_i^2 + \lambda_3 NC_i^3 + \lambda_4 NC_i^4 + \epsilon_i, \ \forall j = 1, 2 \tag{8.2}$$

where :

- j is an indicator for whether the cost is for consultation with generalists or specialists;
- $cost\_consultation_{i,j}$ is the annual cost of consultations for the individual i;
- NC_i is the number of consultations for the individual i;
- NC_i^2 is the number of consultations for the individual i raised to the power of 2;
- NC_i^3 is the number of consultations for the individual i raised to the power of 3;
- NC_i^4 is the number of consultations for the individual i raised to the power of 4;
- ϵ_i is a random term specific to the individual i.

We estimate these two models for men and for women separately, for a total of four models. They are estimated among individuals who visited a doctor at least once in the last 12 months. Table 8.2 presents the estimate of the determinants of the annual cost of consultations.

	GP consultations	
	Men	Women
Number of consultations	50.48885***	45.83090***
(Number of consultations)^2	0.56933***	1.11615***
(Number of consultations)^3	−0.01681***	−0.04247***
(Number of consultations)^4	0.00006	0.00034***
Number of observations	92,624	143,107
Average cost ($)	218	228
	Specialist consultations	
	Men	Women
Number of consultations	82.95037***	78.75006***
(Number of consultations)^2	−0.10072	0.33086*
(Number of consultations)^3	0.00189	−0.01997***
(Number of consultations)^4	−0.00007	0.00019***
Number of observations	78,634	127,092
Average cost ($)	451	399
Legend	* p <0.10 ; ** p <0.05 ; *** p <0.001	

Table 8.2: Average marginal effect of each variable on the annual cost of consultations

8.3.3 Long-term care facilities

The cost of long-term care facilities is not used in this article. For detailed information on the cost for long-term care facilities is estimated, see section 8.3.3 of the full technical documentation.

8.3.4 Home care services

The cost of home care services is not used in this article. For more information on how the cost of home care services is estimated, see section 8.3.4 of the full technical documentation.

8.4 Aggregate costs

In order to obtain aggregate costs for hospitalizations and physicians that are in line with actual government expenditure on those items, we need to rescale the total expenses obtain with our cost models, for a few different reasons. First, as explained in section 8.1, the costs we obtain for hospitalizations are relative costs. Second, the question we use from the NPHS does not ensure we obtain a total number of physician consultations that reflects the number actually charged by doctors. For instance, a radiologist analyzing an x-ray will charge the government, but a survey respondent would surely not count this separately when asked "how many times did you see a doctor?". The assumption underlying the scaling is that even if the number of consultations is not the same in administrative data and in survey data, the relative use is.

For scaling we use CIHI data on public health expenditures in Canadian Institute for Health Information (2014). Table 8.3 shows the aggregate costs obtained in CIHI and from COMPAS and the ratios used to scale up the aggregate costs from COMPAS. Because CIHI does not separately identify the cost for generalists and specialists, we use total billing for doctors as reported by RAMQ (Régie de l'assurance maladie du Québec (2012)) to distribute the "physicians" item from CIHI.

	CIHI/RAMQ		COMPAS		Ratio
Hospital	9 304.5	66,7%	5 831.0	84.1%	1.6
Generalist	1 785.1	12,8%	744.8	10.7%	2.4
Specialist	2 851.4	20,5%	360.2	5.2%	7.9
Total	13 941.0	100.0%	6 936.0	100.0%	2.0

Table 8.3: Aggregate expenses on hospitals and physicians in 2012 according to CIHI/RAMQ and COMPAS, and scaling ratios

Chapter 9

Uncertainty

Uncertainty in COMPAS is managed with a Monte-Carlo setup. Multiple sources of uncertainty are take into consideration. We take into account the stochastic (first-order) nature of microsimulation as well as parameter uncertainty (Briggs et al., 2012). Individual heterogeneity is accounted for by the integration of individual characteristics in every econometric model used in COMPAS.

9.1 Types of uncertainty

We re-estimate each transition model (as described in chapter 4) 100 times using a bootstrap approach. In order to take into account the joint distribution of parameters both intra-model and inter-model, we draw with replacement from our datasets and re-estimate each transition model, completing this procedure 100 times. This provides a good estimation of the parameter distribution.

For each joint set of parameters, we then run the simulation 50 times to take into account stochastic uncertainty. We retain the mean of these 50 runs of the simulation as the result for each set of parameters. We obtain 100 sets of results that therefore incorporate first-order, second-order, and heterogeneity uncertainty.

We also integrate two other sources of uncertainty. First, we generate 100 initial databases to take into account the random process of imputation of some variables, as mentioned in chapter 3. Second, we also recalculate the joint distribution among each draw of the entering cohorts.

9.2 Presentation of the uncertainty

We present the 5th and 95th percentiles of the different sets of parameters as an outcome's 90% uncertainty (or confidence) interval. We chose to present these because of the asymmetrical distribution of the estimates around the mean outcome.

9.3 Summary

The whole process to account for uncertainty can therefore be summarized by these 5 steps for a particular scenario:

- 100 bootstrap sets of parameters are estimated.
- For each set of parameters, a simulation is run 50 times to account for stochastic uncertainty.
- Each run uses a different initial dataset and different entering cohorts. These vary as a function of the inherent randomness of the imputation processes.
- We obtain 50 outcome values for each of the 100 sets of parameters, for a total of 5,000 values.
- The mean is reported for each outcome, along with the 5th and 95th percentiles as uncertainty (or confidence) interval computed for each set of parameters.

Bibliography

Bohnert, N., Chagnon, J., Coulombe, S., Dion, P., and Martel, L. (2015). Population Projection for Canada (2013 to 2063), Provinces and Territories (2013 to 2038): Technical Report on Methodology and Assumptions. Technical report, Statistics Canada.

Boulenger, S. and Castonguay, J. (2012). Portrait de la rémunération des médecins de 2000 à 2009. *Scientific Series*, Centre interuniversitaire de recherche en analyse des organisations (CIRANO) - 2012s-12.

Briggs, A. H., Weinstein, M. C., Fenwick, E. A., Karnon, J., Sculpher, M. J., and Paltiel, A. D. (2012). Model parameter estimation and uncertainty: A report of the ispor-smdm modeling good research practices task force-6. *Value in Health*, 15(6):835 – 842.

Cameron, A. and Trivedi, P. (2005). *Microeconometrics: Methods and Application*. New York: Cambridge University Press.

Canadian Cancer Society's Advisory Committee on Cancer Statistics (2015). Canadian cancer statistics 2015. *Canadian Cancer Society*.

Canadian Institute for Health Information (2014). National Health Expenditure Trends, 1975 to 2014. Technical report.

Cancer Research UK (n.d.). Lung cancer survival statistics Read more at http://www.cancerresearchuk.org/health-professional/cancer-statistics/statistics-by-cancer-type/lung-cancer/survivalrVU6y20MxJ3HGRm1.99.

Frees, E., Gao, J., and Rosenberg, M. (2011). Predicting the frequency and amount of health care expenditures. *North American Actuarial Journal*, 15(3):377–382.

Goldman, D., Shekelle, P., Bhattacharya, J., Hurd, M., Joyce, G., Lakdwalla, D., Matsui, D., Newberry, S., Panis, C., and Shang, B. (2005). Health status and medical treatment of the future elderly. Technical report, RAND Corporation.

Human Mortality Database (2005). Complete data series. Technical report.

Régie de l'assurance maladie du Québec (2012). Table sm.22. Technical report, Régie de l'assurance maladie du Québec.

Statistics Canada (2008). Canadian Community Health Survey - healthy aging. Data files, Statistics Canada.

Statistics Canada (2010). Canadian Community Health Survey, 2010, Wave 5.1, Annual component. Data sets, Statistics Canada.

Statistics Canada (2011). 2011 Census of Population, Statistics Canada catalogue no. 98-313-xcb2011024. Technical report, Statistics Canada.

Statistics Canada (2012a). National Population Health Survey—Household component — longitudinal (NPHS). Technical report.

Statistics Canada (2012b). National Population Health Survey, 1994-2010, waves 1-9, Household component- longitudinal. Data sets.

Statistics Canada (2015). Population projections for Canada (2013 to 2063), provinces and territories (2013 to 2038). Technical report, Statistics Canada, National Population Projections team.

Statistics Canada (n.d.a). Estimates of population, by age group and sex for July 1, Canada, provinces and territories annual (persons unless otherwise noted). *CANSIM (database). Last updated September 29, 2015.*

Statistics Canada (n.d.b). Table 052-0005: Projected population, by projection scenario, age and sex, as of july 1, canada, provinces and territories. *CANSIM (database). Last updated October 17, 2014.*, http://www5.statcan.gc.ca/cansim/a26?lang=eng&id=520005 (Accessed: 2016-05-29).

Sueyoshi, G. T. (1995). A class of binary response models for grouped duration data. *Journal of Applied Econometrics*, Vol. 10, 1995,(No. 4):pp. 411–431.
